# Alcohol dehydrogenases AdhE and AdhB with broad substrate ranges are important enzymes for organic acid reduction in *Thermoanaerobacter* sp. strain X514

**DOI:** 10.1186/s13068-021-02038-1

**Published:** 2021-09-25

**Authors:** Lisa Hitschler, Laura Sofie Nissen, Michelle Kuntz, Mirko Basen

**Affiliations:** 1 Molecular Microbiology and Bioenergetics, Institute of Molecular Biosciences, Johann Wolfgang Goethe University Frankfurt/Main, Max-von-Laue Str. 9, 60438 Frankfurt/Main, Germany; 2 Present Address: Department of Membrane Biochemistry, Life and Medical Sciences (LIMES) Institute, University of Bonn, Carl-Troll-Straße 31, 53115 Bonn, Germany; 3 Present Address: Microbiology, Institute of Biological Sciences, University of Rostock, Albert-Einstein Str. 3, 18059 Rostock, Germany; 4 Present Address: Interfaculty Institute for Microbiology and Infection Medicine Tübingen, University of Tübingen, Auf der Morgenstelle 24, 72076 Tübingen, Germany

**Keywords:** *Thermoanaerobacter*, Thermophile, Ethanol fermentation, Organic acid reduction, Alcohol dehydrogenase, Aldehyde:ferredoxin oxidoreductase

## Abstract

**Background:**

The industrial production of various alcohols from organic carbon compounds may be performed at high rates and with a low risk of contamination using thermophilic microorganisms as whole-cell catalysts. *Thermoanaerobacter* species that thrive around 50–75 °C not only perform fermentation of sugars to alcohols, but some also utilize different organic acids as electron acceptors, reducing them to their corresponding alcohols.

**Results:**

We purified AdhE as the major NADH- and AdhB as the major NADPH-dependent alcohol dehydrogenase (ADH) from the cell extract of the organic acid-reducing *Thermoanaerobacter* sp. strain X514. Both enzymes were present in high amounts during growth on glucose with and without isobutyrate, had broad substrate spectra including different aldehydes, with high affinities (< 1 mM) for acetaldehyde and for NADH (AdhE) or NADPH (AdhB). Both enzymes were highly thermostable at the physiological temperature of alcohol production. In addition to AdhE and AdhB, we identified two abundant AdhA-type ADHs based on their genes, which were recombinantly produced and biochemically characterized. The other five ADHs encoded in the genome were only expressed at low levels.

**Conclusions:**

According to their biochemical and kinetic properties, AdhE and AdhB are most important for ethanol formation from sugar and reduction of organic acids to alcohols, while the role of the two AdhA-type enzymes is less clear. AdhE is the only abundant aldehyde dehydrogenase for the acetyl-CoA reduction to aldehydes, however, acid reduction may also proceed directly by aldehyde:ferredoxin oxidoreductase. The role of the latter in bio-alcohol formation from sugar and in organic acid reduction needs to be elucidated in future studies.

**Supplementary Information:**

The online version contains supplementary material available at 10.1186/s13068-021-02038-1.

## Background

Short-chain alcohols such as ethanol or isobutanol represent valuable next-generation biofuels which may be produced by microorganisms as biocatalysts (in vivo) or by enzymatic pathways derived from microbes (in vitro). However, in-depth understanding of the physiology of the microorganisms and of the underlying biochemical pathways is essential, in order to improve to the biological and technological productivities, or even to transfer the biochemistry to different microbial production platforms. The biochemistry of alcohol production by thermophilic microorganisms is of high interest, since fermentations at elevated temperatures harbor inherent technical and economic advantages such as faster reaction rates, lower risk of contaminations and lower costs for cooling the self-heating bioreactors [1]. Species of the genus *Thermoanaerobacter* have been described as thermophilic, chemoorganotrophic sugar-utilizing microorganisms, producing a wide range of fermentation products such as acetate, lactate, CO_2_, H_2_ or ethanol [2], with the notable exception of the (homo)acetogen *T. kivui* [3–5]. In many *Thermoanaerobacter* species though, ethanol is the major product. In particular, species of a subgroup, Clade 1, were described as extremely efficient in ethanol formation from sugar [6, 7], yet reaching lower titers than commercial ethanol producers such as yeasts or the bacterium *Zymomonas mobilis* [8]. We recently found that a variety of *Thermoanaerobacter* species not only produced ethanol from sugars, but also took up and subsequently reduced organic acids to their corresponding alcohols, e.g., isobutyrate to isobutanol [9]. Compared to ethanol, isobutanol is a better biofuel, since it has a higher volumetric energy content, a lower volatility and a lower hygroscopicity than ethanol [10]. Again, species of Clade 1, such as *Thermoanaerobacter* sp. strain X514, *T. pseudethanolicus* and *T. brockii* ssp. *finnii* produced the highest alcohol concentrations. More evidence of organic acid reduction to alcohols has since been put forward for other *Thermoanaerobacter* strains (AK85 and AK152) [11, 12].

**Fig. 1:**
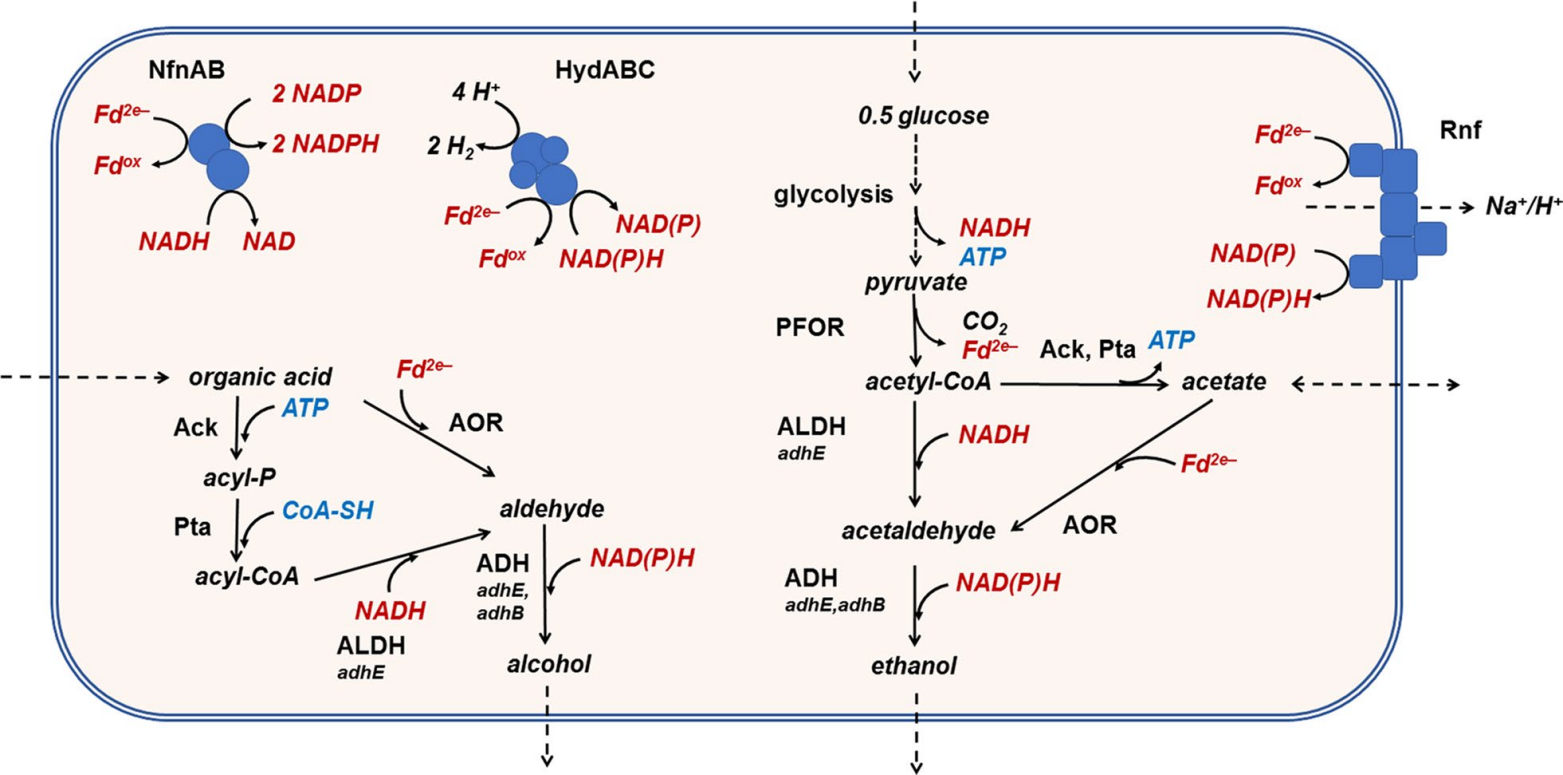
Model and potential role of key enzymes in alcohol production and redox metabolism of ferredoxin or nicotinamide cofactors in *Thermoanaerobacter* sp. strain X514. pyruvate ferredoxin oxidoreductase (PFOR), aldehyde ferredoxin oxidoreductase (AOR), aldehyde dehydrogenase (ALDH), alcohol dehydrogenase (ADH), ferredoxin (Fd), acetyl-CoA synthetase (Acs), phosphotransacetylase (Pta); acetate kinase (Ack), membrane-bound ferredoxin:NAD(P) oxidoreductase (Rnf), NADH-dependent reduced ferredoxin:NADP oxidoreductase (Nfn), electron-confurcating Fe–Fe hydrogenase (HydABCD)

The biochemical pathway of organic acid reduction is currently under investigation. It may proceed via the same route as ethanol is produced fermentatively from sugars. However, despite significant efforts from different laboratories, even the pathway of ethanol formation from sugar in *Thermoanaerobacter* spp. has also not been ultimately resolved [13, 14]—and more importantly, the involved enzymes may differ among species [9]. Two general pathways have been proposed from acetyl-coenzyme A to ethanol in *Thermoanaerobacter* spp. (Fig. 1): (a) a two-step nicotinamide NAD(P)H-dependent reduction via the intermediate acetaldehyde [13] involving an acetyl-CoA reducing aldehyde dehydrogenase (ALDH) or (b)—in some species of Clade 1—the ATP-conserving conversion to acetate, which is then further reduced by the ferredoxin-dependent direct reduction of acetate to acetaldehyde by aldehyde:ferredoxin oxidoreductase (AOR) [9]. The reducing equivalents are provided by glycolysis and by pyruvate oxidation in form of reduced nicotinamide adenine dinucleotide cofactors and reduced ferredoxin, respectively. Both pathways theoretically also operate with externally added organic acids as electron acceptors. In case of (a), the two-step nicotinamide NAD(P)H-dependent reduction, in a reversal of acetate forming reactions, the acid must be phosphorylated to the acyl phosphate by acetate kinase first, and then converted to an acyl-CoA by phosphotransacetylase. The correlation of *aor* genes and AOR activity in strains with high rates of organic acid reduction such as *Thermoanaerobacter* sp. strain X514 (Clade 1) points towards an involvement of AOR in alcohol production by direct acid reduction to an aldehyde [9], however, indisputable evidence has not been brought forward yet.

In both pathways, alcohol dehydrogenases (ADHs) are involved in the nicotinamide-dependent reduction of acetaldehyde. In this study, we aimed to identify and characterize the most important ADHs in the efficient organic acid reducer *Thermoanaerobacter* sp. strain X514. Its genome contains nine *adh* genes [15–17], and it is unclear which of these are involved in ethanol formation from sugars and in organic acid reduction to alcohols. Based on genomic and transcriptomic studies performed with *Thermoanaerobacter* sp. strain X514, Lin et al*.* (2011) suggested a crucial role for the ADH encoded by Teth514_1935 [16], while Hemme et al*.* (2011) suggested that AdhB likely catalyzed the aldehyde reduction [17]. Due to the lack of tools enabling genetic modification of the (*aor* containing) *Thermoanaerobacter* sp. strain X514, we chose to purify the dominant nicotinamide-dependent acetaldehyde reducing activities from cells grown with glucose. We analyzed *adh* gene expression and protein production and biochemically characterized the major ADHs towards their substrate spectra, substrate affinities and thermostabilities.

## Results and discussion

### Efforts towards genetic modification of *Thermoanaerobacter* sp. strain X514

Nine genes encoding for putative ADHs and one *aor* gene (Teth514_1380) are present in the genome of *Thermoanaerobacter* sp. strain X514, but it remains unclear which of those genes play a role in alcohol production, from sugar as well as in organic acid reduction[9]. One of those genes is annotated as an ‘ADH, zinc-binding domain protein’ (Teth514_0653), one as ‘short-chain dehydrogenase/reductase SDR’ (Teth514_1808), one other as a ‘GroES domain protein’ (Teth514_1882) and the remaining six genes as ‘iron-containing ADH’ (Teth514_0145, Teth514_0241, Teth514_0564, Teth514_0627, Teth514_0654, Teth514_1935). The major aim of our study was to shed light on the role of ADHs in *Thermoanaerobacter* sp. strain X514, in sugar fermentation to ethanol and in organic acid reduction to alcohols. Initially, we aimed towards a genetic approach, including the deletion of *adh* genes and *aor* from the genome. The strain has been reported to be transformed with plasmid DNA, using electroporation or ultrasound [18]. We succeeded in transforming the strain by supplying plasmid DNA in different growth phases. *Thermoanaerobacter* sp. strain X514 was naturally competent for DNA uptake during a very brief period in the mid-exponential growth phase around an OD_600_ of 0.52 (Additional file 1: Fig. S1). The transformation frequency of up to 2 × 10^–5^ compares to other *Thermoanaerobacter* strains [19], while the transformation efficiency of 470 colonies per µg plasmid DNA is half of what has been reported for the related *T. kivui* [20]. However, *Thermoanaerobacter* sp. strain X514 resisted our efforts to insert DNA into its chromosome; and to our knowledge, genomic modifications have not been reported. While in the past decade, progress with genetic methods for some *Thermoanaerobacter* species such as *T. mathranii* [21]*, T. ethanolicus* [22] or *T. kivui* [20] has been reported, the difficulty to develop genetic methods is well-documented for clostridial anaerobes [23].

### Purification of the major alcohol and aldehyde dehydrogenases AdhB and AdhE

Consequently, we chose a biochemical approach and purified the abundant NAD(P)H-dependent ADHs from the cell extracts of *Thermoanaerobacter* sp. strain X514. Cell-free extract of cells grown on 25 mM glucose carried out NADH-dependent ALDH as well as NADH-dependent and NADPH-dependent ADH activity. ALDH was measured as coenzyme A (CoA)-dependent reduction of NAD^+^ with acetaldehyde, while ADH was measured as acetaldehyde-dependent oxidation of NAD(P)H. The cell-free extract contained NADH-dependent ADH activity (1.2 U mg^−1^), and a comparably high NADPH-dependent ADH activity of 2.5 U mg^−1^, the latter being higher than in the related *T. pseudethanolicus* [0.7 U mg^−1^, 24]. The dominant enzyme responsible for NADH-dependent ADH activity was purified using ~ 15 g of wet cells in three consecutive chromatographic steps, anion exchange chromatography (Q-Sepharose), hydrophobic interaction chromatography (phenyl-Sepharose) and size exclusion chromatography (Superose 6) as described below (Table 1). The enzyme responsible for NADPH-dependent ADH activity was assayed as NADPH-dependent acetaldehyde reduction and purified using the same three consecutive chromatographic steps (Table 1), with both enzymes, the NADPH-dependent ADH and the NADH-dependent ALDH/ADH being separated in the last chromatographic step.

**Table 1:** Purification of the major NADH-dependent alcohol dehydrogenase AdhE of *Thermoanaerobacter* sp. strain X514. NADH-dependent ADH activity was measured in 50 mM Tris buffer (pH 7.5) supplemented with 2 mM DTE and 4 µM resazurin as NADH (0.5 mM)-dependent reduction of acetaldehyde (10 mM) at 65 °C

Purification step	Total protein [mg]	Specific activity [U mg^−1^]	Total activity [U]	Yield [%]	Purification [fold]
Cell-free extract	1423	1.2	1734	100.0	1.0
Cytoplasm	1196	1.8	2183	125.9	1.5
Q-Sepharose	877	2.7	2323	134.0	2.2
(NH_4_)_2_SO_4_ precipitation	511	4.7	2413	139.2	3.9
Phenyl-Sepharose	220	7.3	1602	92.4	6.0
Superose 6	2.8	61.2	173	9.9	50.2

The decrease in absorption of NADH was followed at 340 nm. One unit represents one µmol of acetaldehyde reduced per minute. The major ALDH activity (CoA and NAD^+^-dependent oxidation of acetaldehyde) co-purified due to the bifunctionality of AdhE (Additional file 1: Table S1)

**Fig. 2:**
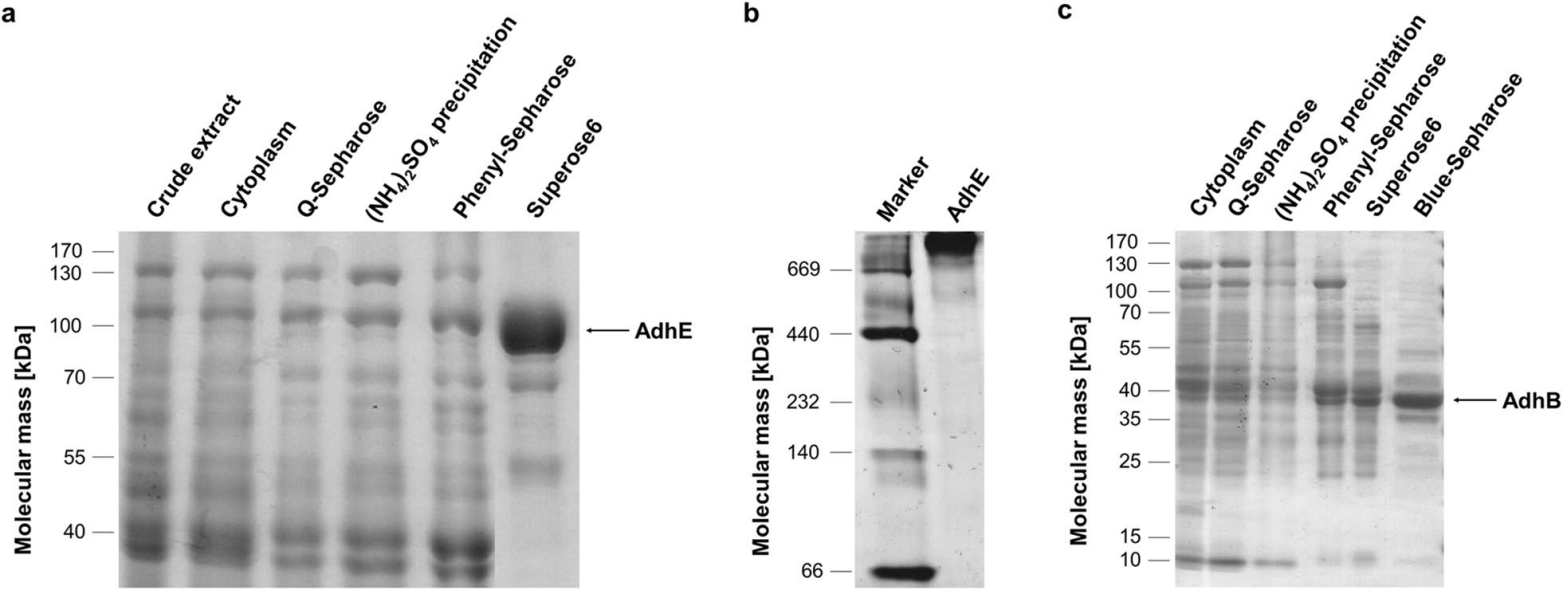
Purification and identification of the major NADH or NADPH-dependent ADH of *Thermoanaerobacter* sp. strain X514, AdhE and AdhB, respectively. **a** Denaturing SDS polyacrylamide gel electrophoresis of the fractions during isolation of AdhE (abundant from *Thermoanaerobacter* sp. strain X514. **b** The purified AdhE was separated by native PAGE under anaerobic conditions. **c** Fractions from the different purification steps of AdhB were separated by SDS-PAGE. 10 μg protein per lane; stained with Coomassie Brilliant Blue

NADH-dependent ADH activity was enriched 50-fold with a specific activity of 61.2 U mg^−1^ (Table 1). SDS polyacrylamide gel electrophoresis (PAGE) of the purified sample showed one major protein with an apparent molecular mass of 100 kDa (Fig. 2A), corresponding to the expected mass of AdhE (96.9 kDa). Liquid chromatography–mass spectrometry (LC–MS) analysis identified the enzyme responsible for NADH-dependent ALDH/ADH activity as an iron-containing ADH (ABY91935.1). This protein is encoded by the gene Teth514_0627 which shares 100% query cover and 97% identity with a bifunctional ALDH/ADH known as AdhE of the strains *T. ethanolicus*, *T. wiegelii* and *T. mathranii*. Furthermore, the putative AdhE of *Thermoanaerobacter* sp. strain X514 is 86% similar to the AdhE of *Thermoanaerobacterium saccharolyticum* and 51% similar to AdhE of *Escherichia coli*. Since the enzyme is bifunctional, ALDH activity co-purified with the NADH-dependent ADH activity, i.e., coenzyme A (CoA)-dependent reduction of NAD^+^ with acetaldehyde as substrate was enriched 82-fold, with a specific activity of 9.7 U mg^−1^ (Additional file 1: Table S1). Since other ALDH activity in the crude extract was negligible, AdhE is the major ALDH in *Thermoanaerobacter* sp. strain X514. Size-exclusion chromatography was the decisive purification step here (Table 1), unlike what has been observed for the purification of the ALDH from *T. pseudethanolicus* and from *A. woodii* where anion exchange chromatography led to a sixfold activity enrichment [24, 25]. In native PAGE under non-denaturing conditions, the purified AdhE was visible in the high molecular range (Fig. 2B), indicating that AdhE from *Thermoanaerobacter* sp. strain X514 forms large polymers. The polymeric structure of AdhE has already been reported for proteins isolated from the bacteria *E. coli*, *Acetobacterium woodii* and *Geobacillus thermoglucosidasius* [25–27].

The second purified enzyme was the sole major NADPH-dependent ADH, despite a loss in total activity of > 90% in this size-exclusion step, no other major activity eluted from on the column. In the last chromatographic step, the enzyme was then further purified using a HiTrap Blue HP column (Fig. 2C), reaching a specific activity of 35.2 U mg^−1^ (Table 2). Separation of the purified sample using SDS-PAGE yielded two proteins with apparent molecular masses of 38 kDa and 36 kDa, respectively (Fig. 2C). Both proteins were identified by LC–MS analysis as a ‘zinc-binding domain ADH’ (ABY91961.1) being encoded by the gene Teth514_ 0653 and sharing 98–96% similarity to AdhB from *T. mathranii*, *T. kivui*, *T. wiegelii*, *T. ethanolicus* and *T. pseudethanolicus*. The expected mass of AdhB is 37.8 kDa. Therefore, the smaller 36–kDa protein found in SDS-PAGE could be a degradation product of AdhB.

**Table 2:** Purification of the major NADPH-dependent alcohol dehydrogenase AdhB of *Thermoanaerobacter* sp. strain X514

Purification step	Total protein [mg]	Specific activity [U mg^−1^]	Total activity [U]	Yield [%]	Purification [fold]
Cell-free extract	512	2.5	1293	100	1.0
Cytoplasm	449	2.6	1150	89	1.0
Q-Sepharose	337	4.8	1599	123	1.9
(NH_4_)_2_SO_4_ precipitation	268	6.6	1772	137	2.6
Phenyl-Sepharose	86.8	14.8	1284	99	5.8
Superose 6	9.2	13.3	122	9.4	5.2
Blue-Sepharose	2.5	35.2	87.3	6.8	13.8

NADPH-dependent ADH activity was measured in 50 mM Tris buffer (pH 7.5) supplemented with 2 mM DTE and 4 µM as NADPH (0.5 mM)-dependent reduction of acetaldehyde (10 mM) at 340 nm. The decrease in absorption of NADPH was followed at 340 nm. One unit represents one µmol of acetaldehyde reduced per minute

### Two additional, highly expressed alcohol dehydrogenases identified by qRT-PCR

To investigate whether one or several of the genes encoding other putative ADHs were additionally expressed during growth on glucose, the transcript abundance of the genes in cells grown on glucose was compared to the transcript abundance of housekeeping genes using qRT-PCR. Additionally, the transcript abundance of the *adhs* from cells grown on glucose was compared to that from cells grown on glucose plus isobutyrate, to see whether any *adh* may be upregulated during organic acid reduction. Among the organic acids isobutyrate had been shown to be reduced to its corresponding alcohol, isobutanol, at the highest rate by *Thermoanaerobacter* sp. strain X514 [9].

**Fig. 3:**
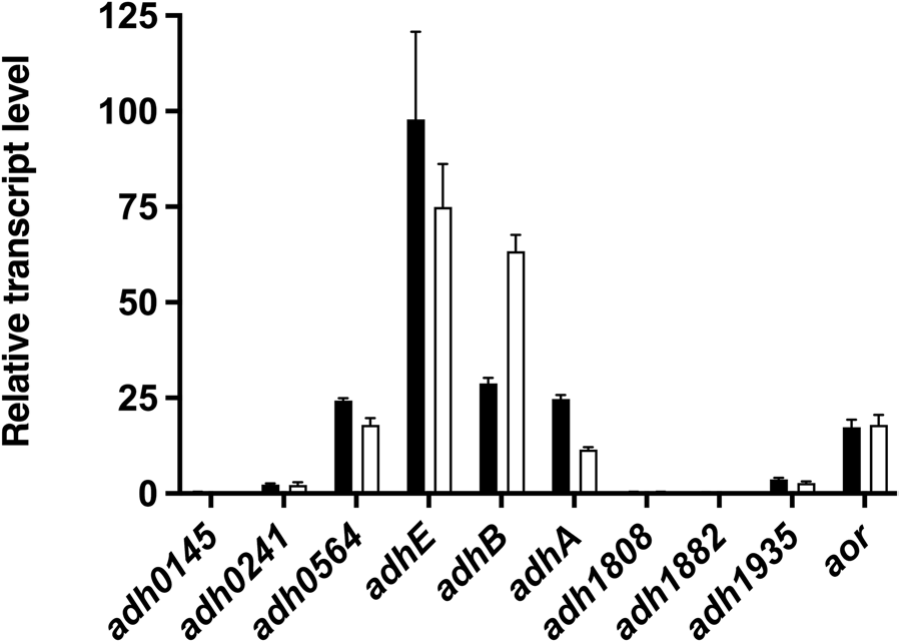
Expression levels of the putative *adh* genes (with their corresponding gene number) and the *aor* gene (Teth514_1380) of *Thermoanaerobacter* sp. strain X514 during growth on 25 mM glucose (black bars) and 25 mM glucose plus 50 mM isobutyrate (white bars). The transcript abundance of the 9 *adh* genes and the *aor* gene was analyzed with qRT-PCR, and is given relative to the abundance of the gene encoding for the DNA gyrase *gyrA* (Teth514_0010) as housekeeping gene (i.e., relative transcript level of *gyrA* equals 1). Teth514_0627, *adhE*; Teth514_0653, *adhB*; Teth514_0654, *adhA*. The average and standard deviation of three independent replicates are shown

Four *adh* genes and the *aor* gene putatively encoding an aldehyde:ferredoxin oxidoreductase (AOR) were highly expressed during growth on glucose (25 mM) and during growth on glucose (25 mM) plus isobutyrate (50 mM) (Fig. 3). Compared to the housekeeping gene encoding DNA gyrase *gyrA* (Teth514_0010), the relative mRNA abundance of the gene Teth514_0627 (*adhE*) was 98 or 75 times as high when grown on glucose or glucose plus isobutyrate, respectively. Interestingly, the transcription level of the gene encoding AdhB, Teth514_0653 (*adhB*) of glucose plus isobutyrate-grown cells was slightly higher compared to growth on glucose with mRNA levels of 63 and 28 times as high as *gyrA*, respectively. In genetic proximity to *adhB* is the gene Teth514_0654 encoding an iron-containing ADH which shares 91% identity with Teth514_0564. Both genes were highly expressed with similar abundances under the studied growth conditions. The protein encoded by Teth514_0654 is 98–99% similar to an iron-containing ADH from *T. brockii*, *T. pseudethanolicus* and *T. mathranii* known as AdhA. Hemme et al*.* also reported that the same three *adh* genes (*adhA, adhB* and *adhE*) were expressed in *Thermoanaerobacter* sp. strain X514 grown on xylose suggesting that the enzymes encoded by these genes are key enzymes involved in ethanol production [17]. In contrast, the expression level of the other five genes (Teth514_0145, Teth514_0241, Teth514_1808, Teth514_1882, Teth514_1935) putatively encoding ADHs as well, is comparably low. Therefore, it is unlikely that they have crucial function in ethanol formation from glucose (and organic acid reduction), somewhat contrary to the conclusion of Lin et al*.* who hypothesized a role for the ADH encoded by Teth514_1935, at least in ethanol formation from fructose or xylose [16].

The corresponding proteins AdhA and Adh0564 of the highly expressed *adh* genes, Teth514_0654 and Teth514_0564 were, unlike AdhB and AdhE, not purified from the cell-free extract of *Thermoanaerobacter* sp. strain X514. Therefore, we decided to overproduce AdhA and Adh0564 in *E. coli* for their biochemical characterization. Additionally, AdhB and AdhE, were overproduced in *E. coli* as well, in order to produce polyclonal antibodies. All proteins carried an engineered His_6_-tag, either C-terminally (AdhB-His, AdhE-His, Adh0564-His) or N-terminally (His-AdhA), for purification of the overproduced proteins via affinity chromatography.

### Biochemical and kinetic properties of the alcohol dehydrogenases

The two ADHs purified “natively” from *Thermoanaerobacter* sp. strain X514, AdhE and AdhB, were most abundant during growth on sugars, where ethanol is the only alcohol produced. Since we recently reported that *Thermoanaerobacter* sp. strain X514 reduced externally added organic acids to alcohols, we studied the biochemical properties of the purified enzymes, which may shed light on their putative role in organic acid reduction via aldehydes.

The bifunctional AdhE protein consists of an ADH as well as an ALDH domain. Both domains were found to be active as the purified enzyme catalyzed NAD^+^-dependent oxidation of primary alcohols as well as NAD(H)-dependent aldehyde reduction and oxidation. A broad range of aldehydes were oxidized with highest activities for acetaldehyde and butyraldehyde, but the enzyme also oxidized propionaldehyde and isobutyraldehyde with 81% and 69% of the ALDH activity compared with acetaldehyde, respectively (Table 3). The reaction was strictly dependent on the presence of coenzyme A (CoA) and NAD^+^. Therefore, the enzyme may be involved in the reverse reaction, the reduction of short-chain CoA-esters to their corresponding aldehydes. The purified AdhE also displayed a broad substrate specificity for the ADH domain with high activities for the reduction of acetaldehyde, propionaldehyde and isobutyraldehyde (with 99% and 88% of the activity for acetaldehyde reduction, respectively). Due to this substrate spectrum, AdhE could play a role in the reduction of externally added organic acids in *Thermoanaerobacter* sp. strain X514 to the corresponding alcohols reported by Hitschler et al*.* [9]. High substrate specificities for propionaldehyde and isobutyraldehyde fits to the results obtained from cell suspension experiments [9] where addition of propionate and isobutyrate led to the highest amount of alcohol produced indicating that AdhE may be involved in the second reaction, the reduction of the aforementioned aldehydes to their corresponding alcohols. Acetaldehyde reduction showed an apparent Michaelis–Menten kinetic with *K*_m_ values of 0.71 mM and 0.14 mM for acetaldehyde and NADH (Additional file 1: Figs. S2, S3), respectively, and a *V*_max_ of 52.3 U mg^−1^. Interestingly, the affinity for acetaldehyde of the recombinant AdhE-His was much lower than that of the native protein (apparent *K*_m_ of 80.5 mM, Additional file 1: Fig. S2), but in the same order of magnitude as reported by Loder et al*.* for the recombinant AdhE (22 mM) [28]. Furthermore, the *V*_max_ of the recombinant AdhE was lower with 25.8 U mg^−1^ (Additional file 1: Fig. S2). The reason for that is currently unclear. Potentially, the His-tag affected the tertiary or quaternary structure of the polymers, resulting in lower affinity and *V*_max_ of AdhE-His. It is known that AdhE forms large polymeric structures called spirosomes that recently have been structurally characterized [29, 30]. Spirosome formation is crucial to AdhE activity, however, it affected mainly ALDH activity [29]. We confirmed that AdhE-His was present in polymers as the native protein by native PAGE (400–1000 kDa), however, we did not precisely determine the number of subunits. It might be possible that recombinant AdhE is not present in polymers as large as the native protein (Fig. 2B) when overproduced in *E. coli*, which could change the kinetic properties of the enzyme. Substrate affinity of the native AdhE for isobutyraldehyde was lower, compared to acetaldehyde, with a *K*_m_ of 7.35 mM and a *V*_max_ of 44.8 U mg^−1^ (Additional file 1: Fig. S4). The ethanol and NAD^+^ dependence of the ADH activity was hyperbolic with *V*_max_ of 3.6 U mg^−1^ and *K*_m_ values of 86.2 mM and 0.1 mM, respectively. For ethanol oxidation, the substrate affinity increased when CoA was present (*K*_m_ of 38.5 mM), an effect which has been described before for the AdhE of A*. woodii* [25]. For the recombinant AdhE-His, temperature and pH optimum as well as thermostability were determined. The optimal pH and temperature for NADH-dependent acetaldehyde reduction catalyzed by AdhE were 6.0 and 70 °C. These optima differ from the *T. ethanolicus-*AdhE which showed highest activities at a pH of 8.0 and 60 °C [13]. For the *Thermoanaerobacter* sp. strain X514-AdhE, a high thermostability was observed at 65 °C. The enzyme was activated at 65 °C as the activity increased during the first 90 min of incubation at this temperature and then remained stable for the residual time of the 2-h stability test (Additional file 1: Fig. S5). At 70 °C, 75 °C and 80 °C AdhE half-lives of 83 min, 20 min and 5 min were determined. Loder et al*.* reported a half-life of 673 min at 60 °C for the recombinant *Thermoanaerobacter* sp. strain X514-AdhE, but only a half-life of 11 min at 70 °C (MOPS buffer, pH 7.9) [28]. Similarly to the change in affinity, the reason for a higher thermostability of the recombinant enzyme at 70 °C is currently unclear. In principle, the purified bifunctional ALDH/ADH AdhE had similar properties than other purified AdhEs, and due to its aldehyde-reducing and CoA-dependent aldehyde-oxidizing activity it might be involved in both, the reduction of short-chain aliphatic CoA-ester (including acetyl-CoA) derived from organic acids and the further reduction of the corresponding short-chain aliphatic aldehydes to alcohols.

**Table 3:** Substrate-dependent specific activities of purified AdhE and AdhB of *Thermoanaerobacter* sp. strain X514

	AdhE		AdhB	
Substrate	[U mg^–1^]		[U mg^–1^]	
	NAD^+^		NAD^+^	NADP^+^
Alcohol oxidation*	*− CoA*	* + CoA*		
Ethanol	2.2	3.3	0.1	8.8
2-Propanol	n. d	n. d	5.3	51.4
1-Propanol	2.0	4.8	n. d	n. d
1-Butanol	1.4	2.3	n. d	n. d
2-Butanol	0	n. d	n. d	n. d
2,3-Butanediol	0	n. d	n. d	n. d
*Aldehyde/ketone reduction*	*NADH*	*NADPH*	*NADH*	*NADPH*
Acetaldehyde	46.0	7.0	4.1	33.3
Propionaldehyde	45.3	n. d	0.9	29.9
Isobutyraldehyde	40.6	n. d	0.6	38.0
Butyraldehyde	29.7	n. d	0	24.4
Phenylacetaldehyde	n. d	n. d	0	2.3
Acetone	0	n. d	0.7	29.4
2,3-Butanedione	0	n. d	1.9	34.7
*Aldehyde oxidation (+ CoA)*	*NAD*^*+*^	*NADP*^*+*^	*NAD*^*+*^	*NADP*^*+*^
Acetaldehyde	10.5	1.1	0.1	0
Propionaldehyde	8.4	n. d	n. d	n. d
Isobutyraldehyde	7.3	n. d	n. d	n. d
Butyraldehyde	10.6	n. d	n. d	n. d

Activities were determined in 50 mM Tris buffer (pH 7.5) supplemented with 2 mM DTE and 4 µM resazurin. Alcohol (200–500 mM) or aldehyde (10–50 mM) oxidation was determined with NAD(P)^+^ (2 mM) as electron acceptor, in the presence or absence of coenzyme A (0.2 mM; AdhE). Aldehyde or ketone (10 mM) reduction to alcohols was determined with NAD(P)H (0.5 mM) as electron donor; and absorption at 340 nm was measured. For details, please see “Methods” section. One unit (U) refers to one µmol of NADP^+^ reduced or NAD(P)H oxidized per minute. *n. d.*: not determined

^*^Please note that CoA-dependent alcohol oxidation requires the reduction of 2 mol of NAD(P)^+^

The second abundant Adh purified from *Thermoanaerobacter* sp. strain X514 cell-free extract, AdhB, catalyzed NADPH-dependent reduction of several aldehydes with high activities for acetaldehyde, propionaldehyde and isobutyraldehyde (with 90% and 114% of the activity for acetaldehyde reduction, respectively) (Table 3). Therefore, AdhB could be involved in the reduction of aldehydes (and ketone) from the conversion of externally added organic acids in cell suspension experiments with *Thermoanaerobacter* sp. strain X514 performed by Hitschler et al*.* [9]. Additionally, as expected for a secondary ADH, AdhB was the only Adh that also carried out significant activity towards the ketones acetone and 2,3-butanedione (Table 3). For the NADPH-dependent reduction of acetaldehyde, a *V*_max_ value of 35.6 U mg^−1^ of the purified AdhB was determined. *K*_m_ values for NADPH of 0.04 mM or 0.03 mM (Additional file 1: Fig. S6) were determined for the native and the recombinant version of the enzyme, and the AdhB therefore has a tenfold higher affinity for NADPH than its homologue in *T. ethanolicus* [31]. The apparent *K*_m_ of the native AdhB (0.49 mM) for acetaldehyde was again lower than that of the recombinant enzyme (1.86 mM), as seen with AdhE, though not as drastically changed. Compared to other native secondary ADHs, the affinity was tenfold higher than that of AdhB in *T. pseudethanolicus*, but tenfold lower than that in *T. ethanolicus* (Additional file 1: Fig. S7) [24, 31]. Apart from aldehyde reduction, the enzyme was the only Adh characterized that catalyzed NADP^+^-dependent oxidation of alcohols with a preference for secondary alcohols, which is consistent with substrate specificities determined for *T. pseudethanolicus*-AdhB [24]. NAD(H) could also be used instead of NADP(H) as electron carrier for ADH reactions, but the activity was reduced. In *T. pseudethanolicus* AdhB was characterized as a bifunctional ALDH/ADH [24]. Pei et al. [13] confirmed this property for the AdhB in *T. ethanolicus*, but only for acetyl-CoA reduction under physiological conditions. Deletion studies in *T. ethanolicus* showed that AdhB alone was responsible for the production ethanol from acetyl-CoA in vivo and therefore could not substitute for AdhE [14]. Furthermore, the authors did not measure ALDH activity in enzyme assays with cell-free extracts of the *adhE* deletion mutant. Because AdhB only possesses a GroES-like and a zinc-ADH domain but not an ALDH domain as AdhE, Zhou et al*.* postulated that the weak ALDH activity measured by Pei et al*.* [13] is part of a side activity of the ADH domain [14]. The AdhB purified from *Thermoanaerobacter* sp. strain X514 in this study displays the same domain structure as the *T. ethanolicus-*AdhB and did show only very weak NAD^+^-dependent ALDH activity (Table 2) showing that the enzyme is not truly bifunctional. The catalytic activity of AdhB for NADPH-dependent acetaldehyde reduction (ADH activity) was the highest at a pH of 6.75 and a temperature of 82.5 °C. *T. pseudethanolicus-*AdhB possesses a similar pH optimum of 6.5 but a higher temperature optimum above 90 °C, while *T. ethanolicus-*AdhB reached the highest activity at a pH of 8.7 and a temperature similar to *Thermoanaerobacter* sp. strain X514-AdhB with 80 °C [13, 24]. The acetaldehyde reduction activity of AdhB remained stable over a time period of 3 h at 65 °C (Additional file 1: Fig. S8). The enzyme stability was also high at 80 °C and 85 °C with half-lives of 70 min and 50 min, respectively. At 90 °C, the activity quickly decreased with a half-life of 6 min, so at very high temperatures the enzyme is less stable than the AdhB of *T. ethanolicus* (*T*_1/2_ 60 min at 95 °C) [13].

Both, AdhE and AdhB exhibit biochemical properties that are of high interest towards their use in biotechnological applications, high affinities for aldehydes and NAD(P)H in aldehyde reduction to alcohols, comparably high temperature stability at 65 °C and broad substrate spectra. These properties may be exploited in in vivo applications, using *Thermoanaerobacter* sp. strain X514 or a non-native host as biocatalyst; or in vitro, e.g., together with aldehyde oxidoreductases to produce diverse alcohols from their corresponding acids.

The purified recombinant Adh0564-His and His-AdhA were tested for substrate specificity and enzyme kinetics. AdhA (ABY91962.1) and Adh0564 (ABY91872.1) share 91% identity and displayed similar substrate specificities. Both enzymes were NADPH-dependent and showed very weak activities for oxidation of alcohols (Table 4). ALDH activity measured as NADP^+^-dependent acetaldehyde oxidation was not detected for AdhA and only to a very low extent (0.05 U mg^−1^) for Adh0564. The two ADHs reduced none of the ketones tested (except for a weak activity of Adh0564 with acetone) but a broad range of aldehydes. Adh0564 displayed the highest activities for isobutyraldehyde and propionaldehyde with 252% and 257% of the ADH activity compared to acetaldehyde. The NADPH, acetaldehyde and isobutyraldehyde dependencies of Adh0564 followed a hyperbolic pattern each, with *K*_m_ values of 0.18 mM, 0.13 mM and 1.41 mM (Additional file 1: Fig. S9–11), respectively, and *V*_max_ values of 3.1 U mg^−1^ and 7.6 U mg^−1^ for acetaldehyde and isobutyraldehyde, respectively. Thus, while the specific activity was higher with isobutyraldehyde, the affinity for acetaldehyde was higher than for the longer-chain aldehyde. For butyraldehyde, a Km value in the same range (0.166 mM) for Adh0564, expressed in the hyperthermophilic archaeon *Pyrococcus furiosus*, has been reported by Basen et al*.* before [32].

**Table 4:** Enzymatic activities catalyzed by His-AdhA and Adh0564-His

Substrate	AdhA Specific activity [U mg^−1^]		Adh0564 Specific activity [U mg^−1^]	
*Alcohol oxidation*	*NAD*^*+*^	*NADP*^*+*^	*NAD*^*+*^	*NADP*^*+*^
Ethanol	0.0	0.0	n. d	1.21
2-Propanol	0.0	0.05	n. d	0.0
*Aldehyde/ketone reduction*	*NADH*	*NADPH*	*NADH*	*NADPH*
Acetaldehyde	0.01	1.47	0.29	3.38
Propionaldehyde	0.06	1.23	n. d	8.70
Isobutyraldehyde	0.07	1.23	n. d	8.52
Butyraldehyde	0.09	0.98	n. d	5.21
Phenylacetaldehyde	0.0	0.71	n. d	7.42
Acetone	0.0	0.0	n. d	0.30
2,3-Butanedione	0.0	0.0	n. d	0.0
*Aldehyde oxidation (CoA)*	*NAD*^*+*^	*NADP*^*+*^	*NAD*^*+*^	*NADP*^*+*^
Acetaldehyde	0.0	0.0	n. d	0.05

The His-tagged enzymes were produced in *E.* *coli* and purified by affinity chromatography. Activities were determined in 50 mM Tris buffer (pH 7.5) supplemented with 2 mM DTE and 4 µM resazurin. Alcohol (500 mM) or acetaldehyde (10 mM) oxidation was determined with NAD(P)^+^ (2 mM) as electron acceptor, in the presence or absence of coenzyme A (0.2 mM; AdhE). Aldehyde or ketone (50 mM) reduction to alcohols was determined with NAD(P)H (0.5 mM) as electron donor; and absorption at 340 nm was measured. For details, please see “Methods” section. One unit (U) refers to one µmol of aldehyde reduced or alcohol / aldehyde oxidized per minute. *n. d.*: not determined

AdhA displayed significantly lower aldehyde reduction activities than Adh0564. The enzyme reduced a variety of aldehydes using NADH as well as NADPH as cofactor, but activities for NADH-dependent reduction were lower by a factor of 10–100 (Table 3). Similarly, the AdhA of *T. mathranii* also showed a preference NADPH, but *T. pseudethanolicus-*AdhA (ABY93892.1), which shares 99% identity with *Thermoanaerobacter* sp. strain X514-AdhA, preferred NADH as cofactor [21, 24]. AdhA had a higher affinity for NADPH than Adh0564 with a *K*_m_ value of 0.038 mM (Additional file 1: Fig. S12), but lower affinities for acetaldehyde and isobutyraldehyde with *K*_m_ values of 0.34 mM and 60.28 mM, and lower *V*_max_ values of 1.1 U mg^−1^ and 1.8 U mg^−1^, respectively (Additional file 1: Figs. S13, Fig. S14). The AdhA isolated from *Thermoanaerobacter pseudethanolicus* showed similar affinities for NADPH and acetaldehyde with *K*_m_ values of 0.09 mM and 0.25 mM, respectively, but the *V*_max_ value was higher with 8.8 U mg^−1^ [24]. We cannot exclude, however, that the lower activity of the recombinant *Thermoanaerobacter* sp. strain X514-AdhA described in here is partly due to incomplete folding of the protein during heterologous production in *E. coli*. Both, Adh0564 and AdhA, reached the highest aldehyde-reduction rates at a pH of 8.0 but differed in their temperature optimum. Adh0564 showed the highest activity for acetaldehyde reduction at 90 °C in 50 mM Tris (pH 7.5) buffer. Basen et al*.* [32] observed a similar optimum for Adh0564 at a temperature range of 80–83 °C for butyraldehyde reduction. Adh0564 inactivated slowly at 65 °C, with a half-life of 120 min. At 70 °C, 80 °C, 85 °C and 90 °C half-lives of 18 min, 3 min, 1 min and less than 1 min were observed (Additional file 1: Fig. S15). AdhA displayed a temperature optimum of 75 °C in buffer containing each 25 mM MES, MOPS, Tris and CHES (pH 9.0). Noticeably, AdhA possessed a low temperature stability with half-lives of only 6 and 1.25 min at 60 °C and 65 °C, respectively (Additional file 1: Fig. S16).

### Presence of ADHs in extracts from organic acid-reducing cells

**Fig. 4:**
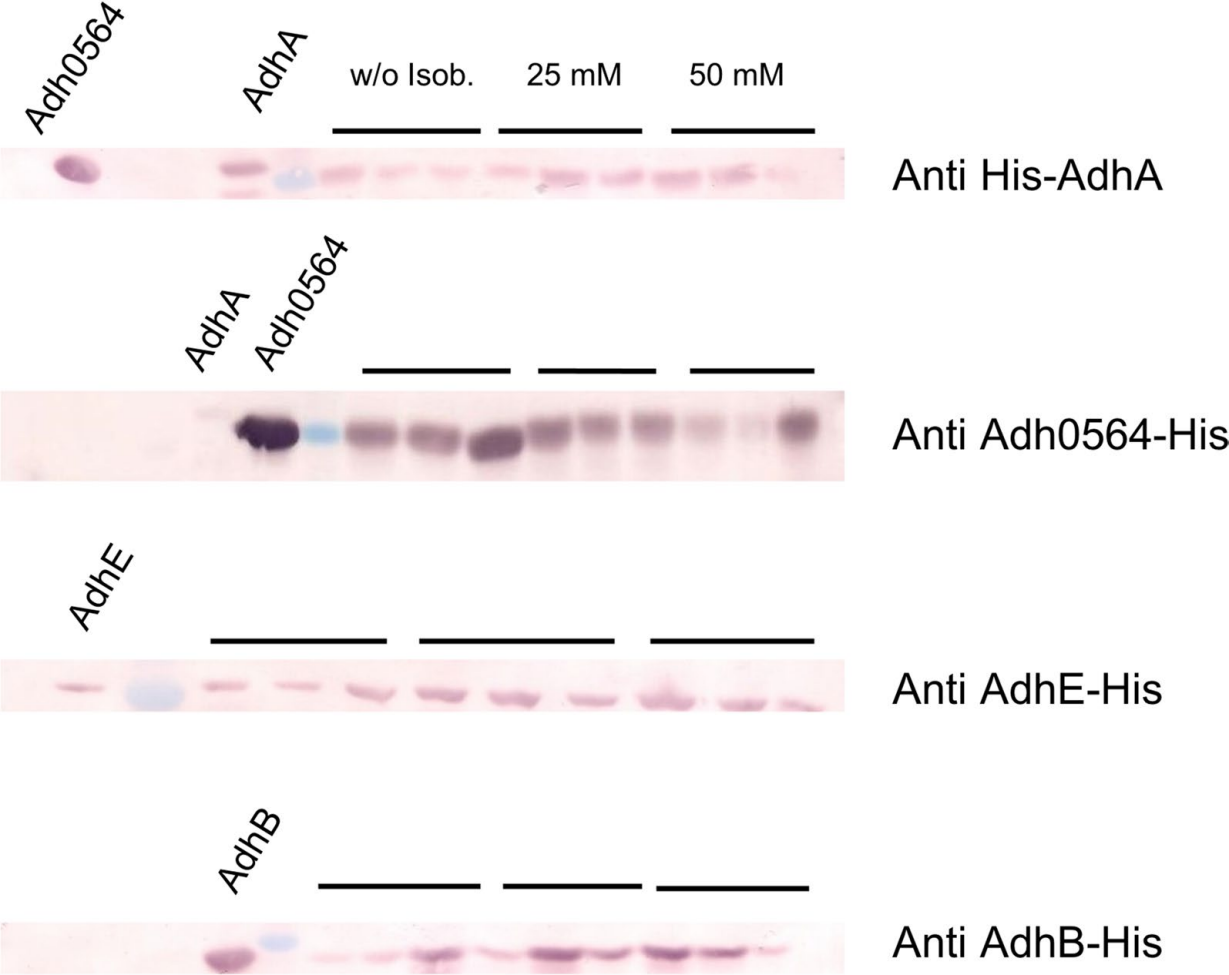
Cellular levels of ADHs in *Thermoanaerobacter* sp. strain X514 grown on glucose (three biological replicates, as indicated by left black bar), on glucose plus 25 mM isobutyrate (as indicated by middle black bar) or on glucose plus 50 mM isobutyrate (as indicated by right black bar). Cell-free extracts (1 μg) were separated via SDS-PAGE. The presence of AdhA, Adh0564, AdhE and AdhB, was determined immunologically with antibodies generated against the His-tagged, affinity-purified proteins. Left lanes, purified proteins as control

To address the question whether the proteins AdhB, AdhE, AdhA and Adh0564 may be involved in ethanol formation from sugars and in reduction of organic acids to alcohols, cells were grown with glucose in the presence or absence of isobutyrate, and the cellular protein levels were monitored by Western blot analysis. Beforehand, the antibodies anti-AdhB-His, anti-AdhE-His, anti-Adh0564-His and anti-His-AdhA were tested for specificity. Anti-AdhB-His and anti-AdhE-His only bound to AdhB or AdhE, respectively. Anti-Adh0564-His and anti-His-AdhA showed cross reactions for AdhA and Adh0564, likely due to the high similarity of these enzymes (91% amino acid sequence identity), though cross-reactivity of Anti-Adh0564-His to His-AdhA was minor. Therefore, it was not possible to determine the presence of AdhA. However, Adh0564 was present in extracts of cells grown with glucose, and in approximately the same concentration range in cells grown with glucose in the presence of 25 mM or 50 mM isobutyrate as electron acceptor (Fig. 4).

AdhE and AdhB were purified as most abundant NADH- and NADPH-dependent ADHs from cell-free extracts of cells grown on glucose. Here, we show that both proteins were also present in approximately the same amounts in the extracts from cells grown under acid-reducing conditions, in the presence of isobutyrate (Fig. 4), underpinning their involvement in alcohol formation from organic acids. The Western blot result therefore reflects the results from the *adh* gene expression levels (Fig. 3), confirming the presence of the enzymes AdhE, AdhB and at least one of the AdhA-type enzymes in the cell-free extracts.

### Suggested roles of the ADHs in organic acid reduction and alcohol formation

Diverse biochemical routes lead from pyruvate to ethanol and from organic acids to their corresponding alcohols (Fig. 1). A major purpose of this study was to shed light on the last step of alcohol formation, the reduction of acetaldehyde to ethanol or other aldehydes to their corresponding alcohols by ADHs. Native purification of NADH or NADPH-dependent ADH activities revealed that AdhE and AdhB are responsible for most of the NADH or NADPH-dependent ADH activity, respectively (Tables 1 and 2, Fig. 2). Both were highly expressed in the presence or absence of isobutyrate as electron acceptor, with *adhB* relative abundance even increasing by a factor of more than 2 in the presence of isobutyrate (Fig. 3). Both proteins were also present in cell extracts in the presence of isobutyrate, as revealed by Western blot analyses (Fig. 4). Both enzymes apparently reduce several aldehydes to their corresponding alcohols with high *V*_max_, differing mainly in their cofactor substrate—while AdhE prefers NADH, AdhB preferentially takes NADPH, both are utilized at high affinities (Table 5). Therefore, we propose that either AdhE or AdhB, or both, catalyze the last step of aldehyde reduction to aldehydes (Fig. 1). From our experiments, we cannot say which of the two enzymes is essential for this step; it may even vary due to the availability of the cofactors (see discussion of model for the redox metabolism below). The role of the other two highly expressed and studied ADHs, AdhA and Adh0564 is less clear. Despite evidence for a relatively high level of expression (Fig. 3), both enzymes were not identified as major ADHs in the cell-free extracts (Table 5). The recombinant versions of both enzymes preferred NADPH over NADH in aldehyde reduction, but both were less active than AdhB, except towards phenylacetaldehyde, where Adh0564 had the highest specific activity. While Adh0564 showed a reduced thermal stability at 65 °C compared to AdhB and AdhE (Table 5), AdhA was very unstable at the physiological temperature optimum of *Thermoanaerobacter* sp. strain X514 of 60 °C [33]. Therefore, we conclude at this point that AdhA is very likely not key to alcohol formation in *Thermoanaerobacter* sp. strain X514, and the role of Adh0564 is unclear, but it is much less likely involved in major aldehyde turnover in vivo than AdhE or AdhB.

**Table 5:**
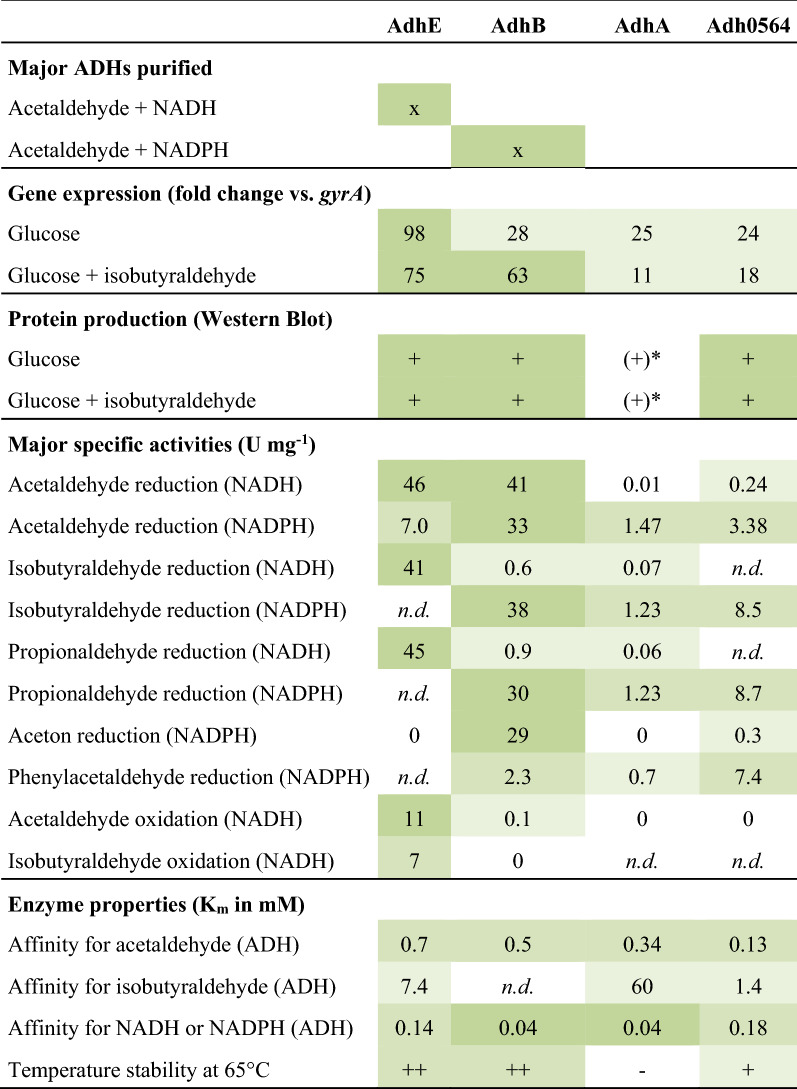
Properties of the four highly expressed alcohol dehydrogenases from *Thermoanaerobacter* sp. strain X514, towards their role in alcohol production

*n.d.* not determined. Protein production: + , present. * Not possible to discriminate between AdhA and AdhA0564 (cross-reactivity of anti-His-AdhA to Adh0564His). Thermostability at 65 °C ++ , no activity decrease for ≧2 h; + , *T*_1/2_ 2 h; –, *T*_1/2_ < 10 min

Gene expression (fold change vs. gyrA): light green >50; dark green >50. Specific activities (U mg^−1^): light green <1, middle green 1-10, dark green >10. Affinities (Km in mM): light green <1, middle green 0,1-1, dark green <0,1.

In addition to its putative involvement as ADH, AdhE is a bifunctional enzyme that is also responsible for the major ALDH activity, the CoA-dependent oxidation of aldehydes, which indicates that in vivo, it may be responsible for acyl-CoA reduction to aldehydes in organic acid reduction and acetyl-CoA reduction to acetaldehyde in ethanol formation from sugar. However, acid reduction to alcohols be may also be initiated by a direct, Fd-dependent reduction catalyzed by AOR (Fig. 1). Here, we show that the gene encoding the enzyme is also highly expressed, though at a slightly lower level than *adhE* (Fig. 3), and the possible involvement of AOR and its implications on redox is discussed below.

The putative importance of both AdhB and AdhE for ethanol formation from sugars (not for organic acid reduction) was described before. All three enzymes, AdhA, AdhB and AdhE have been purified as major ADHs or ALDH, respectively, from sugar-grown *Thermoanaerobacter pseudethanolicus* [24], and differences and similarities in their biochemical properties are described above. Pei et al*.* studied enzymatic properties of the three enzymes from *Thermoanaerobacter ethanolicus* under assumed physiological conditions regarding the concentrations of cofactors, pH and temperature [13]; and the expression of their corresponding genes. They concluded that likely all three enzymes may be involved in acetaldehyde reduction to alcohols, and AdhB or AdhE may catalyze the reduction of the CoA-ester. A complex expression pattern reveals that all three enzymes may also be involved in the reverse reaction, the oxidation of ethanol. After development of genetic tools, in *T. ethanolicus* and *T. mathranii*, the essential role of AdhE for sugar fermentation to ethanol has been shown by genetic experiments [14, 21] likely due to its prevalence as sole major ALDH in the cells, while the role of the NADPH-dependent ADHs AdhB and AdhA is less clear since strains with deletions still produced considerable amounts of ethanol [14, 21]. Interestingly, single deletions of either AdhA and AdhB even increased ethanol yields in *T. ethanolicus* [14], and a double-knockout strain only produced ~ 20% less ethanol. A major difference to *Thermoanaerobacter* sp. strain X514 is that in both latter species, a role for AOR has not been assumed, since the AOR-Adh pathway has been only recently been discussed [32, 34], and the genome of both species did not contain an annotated *aor* gene [14].

From the biochemical data of the purified AdhB and AdhE, we propose two models for alcohol production in *Thermoanaerobacter* sp. strain X514, one involving AdhE and the other involving direct reduction of organic acids by AOR. AdhE is bifunctional and catalyzes NADH-dependent reduction of acetyl-CoA to ethanol. AdhB catalyzes NADPH-dependent reduction of acetaldehyde (Fig. 1). Due to the broad substrate spectrum of AdhE and AdhB regarding the reduction of aldehydes (Table 3), both enzymes may also be involved in the reduction of a variety of organic acids to the corresponding alcohols reported for *Thermoanaerobacter* sp. strain X514 [9]. AdhE could reduce acyl-CoA esters generated by acetate kinase and phosphotransacetylase while AdhB or AdhE could reduce the corresponding aldehydes (Fig. 1).

Since GAPDH in glycolysis is *Thermoanaerobacter* sp. strain X514 is NADH-dependent (data not shown), it remains to be elucidated which enzymes provide NADPH to AdhB. Electrons may be transferred from Fd to NADP^+^ by ferredoxin:NAD(P) oxidoreductases (FNOR). In the genome of *Thermoanaerobacter* sp. strain X514 genes encoding two FNOR enzyme complexes are annotated, the Rnf complex and an electron-bifurcating NADH ferredoxin:NADP^+^ oxidoreductase (Nfn). All six genes (Teth514_079-84) encoding an ion-translocating Rnf complex are present [6, 17], and the genomic organization of the subunits is similar to the acetogen *Acetobacterium woodii.* The protein complex was biochemically characterized in the latter, coupling ferredoxin oxidation and NAD^+^ reduction with the built-up of a Na^+^ ions across the membrane [35, 36]. For *Thermoanaerobacter* sp. strain X514 the activity may be NADP^+^-dependent instead of NAD^+^. In cell-free extracts of *Thermoanaerobacter brockii* FNOR activity was found by Lamed and Zeikus [37], but experimental evidence has not yet been found that the Rnf complex is essential for ferredoxin re-oxidation and for providing reduced NAD(P)H for acetyl-CoA reduction to alcohols. Nfn is the other possible NAD(P)H-producing protein encoded in the genome of *Thermoanaerobacter* sp. strain X514 (Teth514_0651-52). It could be responsible for electron transfer from ferredoxin to nicotinamide cofactors, as Wang et al*.* found in *Clostridium kluyveri* [38]*.* Furthermore, Lo et al*.* reported loss of NADPH-linked ferredoxin oxidoreductase activity when deleting *nfnAB* in *Thermoanaerobacterium saccharolyticum* [39]*.*

Another enzyme which could be involved in organic acid reduction is AOR, replacing the ATP-dependent activation of organic acids and the NADH-dependent ALDH activity carried out by AdhE. The *aor* gene was also highly expressed during growth on glucose and glucose plus isobutyrate (Fig. 1). In cell suspension experiments, *Thermoanaerobacter* strains harboring an *aor* gene were able to reduce a variety of organic acids to the corresponding alcohols using glucose as electron donor. Furthermore, AOR activity in *Thermoanaerobacter* strains carrying a putative *aor* gene was measured, and the specific activity increased when grown in the presence of isobutyrate [9]. Putatively, AOR could catalyze the direct reduction of the acids to the corresponding aldehydes (Fig. 1), however, a detailed biochemical purification and characterization of the enzyme is pending.

## Conclusions

The bifunctional ALDH/ADH AdhE and the secondary ADH AdhB are responsible for most of the NADH and NADPH-dependent ADH activities found in the cell extract of *Thermoanaerobacter* sp. strain X514, respectively. Both AdhE and AdhB were purified from the cell extract, and biochemically characterized. With a broad substrate spectrum for the reduction of different aldehydes and high affinities for aldehydes as well as for nicotinamide cofactors, they are key enzymes in alcohol formation from acetyl-CoA or from organic acids in this organism; and may be biotechnologically exploited in in vitro or in vivo systems for bio-alcohol production. Genes encoding the two primary AdhA-type ADHs, AdhA and Adh0564, were highly expressed, however, they were not purified as major NAD(P)H-oxidizing enzymes from the crude extracts. Their biochemical properties do not support a major role in alcohol production in *Thermoanaerobacter* sp. strain X514. Based on expression data, the remaining 5 ADHs present in the genome are neither involved in ethanol production from sugar nor in alcohol production from organic acids. The redox biochemistry of alcohol metabolism still has to be elucidated, especially regarding the possible involvement of the aldehyde:ferredoxin oxidoreductase, and regarding enzymes providing NAD(P)H from reduced ferredoxin for aldehyde reduction.

## Methods

### Cultivation of *Thermoanaerobacter* sp. strain X514.

*Thermoanaerobacter* sp. strain X514 (ATCC® BAA-938™) was kindly provided by Prof. M. Adams (Department of Biochemistry and Molecular Biology, University of Georgia, Athens, USA). The preparation of anoxic media and the anaerobic cultivation were performed according to Widdel and Bak [40]. The strain was routinely cultivated at 65 °C on modified complex DSMZ medium 516 supplemented with 25 mM glucose, as described previously [9].

### Natural DNA uptake

A fresh culture of *Thermoanaerobacter* sp. strain X514 was inoculated to an OD_600_ of 0.05 in complex medium, as described above, and growth was followed by determination of OD_600_ until the stationary phase was reached. Whenever the OD_600_ increased by 0.1,_._ subsamples were incubated for one hour at 65 °C with 1 µg ml^–1^ DNA of plasmid pIKM1 reported to confer resistance to kanamycin in the related *Thermoanaerobacterium saccharolyticum* [41]. Cells were then embedded in complex medium with 1.5% agar, in the presence of an inhibitory concentration of kanamycin (25 µg ml^–1^), and incubated in a anoxic jar [20] for 5 days at 60 °C.

### Analysis of gene expression levels

50 mL of *Thermoanaerobacter* sp. strain X514 cell culture grown on glucose or glucose and isobutyrate were harvested in late-exponential growth phase (OD_600 nm_ ∼ 0.5–0.7) by centrifugation at 4000×*g*. for 10 min at 4 °C. Cell lysis, total RNA extraction, removal of residual DNA and cDNA synthesis were performed as described previously [42].

The relative transcript level of the nine putative ADH (*adh*) and one aldehyde:ferredoxin oxidoreductase (*aor*) gene was analyzed by qRT-PCR with cDNA as template using a Rotor-Gene RG-3000 qPCR cycler (Corbett Research, Cambridge, United Kingdom) and the Maxima SYBR Green qPCR Master Mix (2×) (Thermo Scientific, Waltham, USA) according to manufacturer’s protocol. The gene for the Gyrase (Teth514_0010) was selected as housekeeping gene. All samples were analyzed in technical triplicates and the primers that were used are listed in Table 4. The thermal cycling conditions were one cycle of initial denaturation at 95 °C for 10 min, 40 cycles of denaturation (95 °C, 15 s), annealing (57 °C, 30 s) and elongation (72 °C, 30 s), followed by a step from 60 to 95 °C (1 °C s^−1^) to establish a melting curve. The resulting data were analyzed with the Rotor Gene Version 6.1 Software (Corbett Research, Cambridge, United Kingdom) and the relative transcript levels were calculated using the 2^−∆∆Ct^ method [43].

### Purification of alcohol dehydrogenases from *Thermoanaerobacter* sp. strain X514

*Thermoanaerobacter* sp. strain X514 was grown under anoxic conditions in 20-L flasks (Glasgerätebau Ochs, Bovenden-Lenglern, Germany) with 25 mM glucose as substrate to an OD_600_ of ~ 0.9. All steps used for purification of the dominant ADHs were performed under strictly anoxic conditions at room temperature in an anaerobic chamber (Coy Laboratory Products, USA) and with buffers containing 2 mM dithioerythritol (DTE) and 4 µM resazurin, as described previously [25]. Cells were harvested (wet weight ~ 15 g), washed with 25 mM Tris buffer (pH 7.5) containing 420 mM saccharose, resuspended in the same buffer containing 3.3 mg mL^−1^ lysozyme and incubated for 1 h at 37 °C. After centrifugation at 9800×*g* for 10 min at 4 °C (Avanti J-25, Beckmann, Glauchau, Germany), the generated protoplasts were resuspended in buffer A (50 mM Tris, 20 mM MgSO_4_, 20% [*v/v*] glycerol, pH 8) including 0.5 mM phenylmethylsulfonyl fluoride (PMSF) and 0.1 mg mL^−1^ DnaseI and passed through a French Press (SLM Aminco; SLM Instruments, USA) at 110 MPa. Intact cells and cell debris were removed by centrifugation at 7,700 × g for 25 min at 4 °C. Membranes were removed by ultracentrifugation at 130,000×*g* for 1 h at 4 °C (Optima L-90 K Ultracentrifuge, Beckmann, Glauchau, Germany).

The enzymes were further purified in three chromatographic steps, anion exchange chromatography (Q-Sepharose), hydrophobic interaction chromatography (phenyl-Sepharose) and size exclusion chromatography (Superose 6). Initially, the cytoplasm with around 600 mg total protein was loaded onto a Q-Sepharose high-performance column (1.6 cm × 11.9 cm) equilibrated with buffer A. A linear gradient from 0 to 1 M NaCl was applied over 90 mL. NADH-dependent ALDH/ADH as well as NADPH-dependent ADH activity was found in the flow-through. Ammonium sulfate was added to a final concentration of 2 M, precipitates were removed from the flow-through by centrifugation at 3400×*g* for 15 min (Eba-200, Hettich Instruments, Tuttlingen, Germany). The supernatant was applied to a phenyl-Sepharose high-performance column (1.6 cm × 15 cm) equilibrated with buffer B (25 mM Tris, 20 mM MgSO_4_, 20% [*v/v*] glycerol, 2 M (NH_4_)_2_SO_4_, pH 8). NADH-dependent ALDH/ADH as well as NADPH-dependent ADH activity eluted at 0–250 mM (NH_4_)_2_SO_4_ in a linear gradient over 120 mL from 2 to 0 M (NH_4_)_2_SO_4_. The fractions with NADH-dependent ALDH/ADH as well as NADPH-dependent ADH activity were pooled and concentrated by using ultrafiltration in VIASPIN tubes (Sartorius Stedim Biotech GmbH, Goettingen, Germany). 100 kDa and 30 kDa filters were used for purification of the dominant NADH-dependent ALDH/ADH and the dominant NADPH-dependent ADH, respectively. Concentrated samples were loaded on a Superose 6 10/300 GL prepacked column (GE Healthcare Life Sciences, Little Chalfont, UK) equilibrated with buffer C (25 mM Tris, 20 mM MgSO_4_, 20% [*v/v*] glycerol, 250 mM NaCl, pH 8) and eluted at a flow rate of 0.3 mL min^−1^. The dominant NADH-dependent ALDH/ADH eluted at 10–15 mL buffer volume and the NADPH-dependent ADH at 15.5–18.5 mL buffer volume. The fractions with NADH-dependent ALDH/ADH and the fractions with NADPH-dependent ADH activity were pooled, respectively. After this step, the pooled fractions with NADH-dependent ALDH/ADH activity showed a protein with apparent homogeneity. The sample containing NADPH-dependent ADH activity was applied to a 5 mL HiTrap Blue HP column (GE Healthcare, Chicago, USA) equilibrated with buffer A. Elution was performed with 500 mM NaCl at a flow rate of 3 mL min^−1^. NADPH-dependent ADH activity was found in the elution volume of 6.5–9.5 mL, fractions were pooled and the pure protein was stored at 4 °C.

### Measurement of enzyme activities

All enzyme assays, unless stated otherwise, were performed in anaerobic cuvettes (Glasgerätebau Ochs, Bovenden-Lenglern, Germany) sealed by rubber stoppers under a N_2_ atmosphere at 65 °C and filled with 1 mL buffer. 50 mM Tris buffer (pH 7.5) supplemented with 2 mM DTE and 4 µM resazurin was used (except for determination of the pH optimum). Enzyme assays for the recombinant AdhB-His, AdhE-His, Adh0564-His and His-AdhA were performed under aerobic conditions and buffers without DTE and resazurin.

Purification of the NADH-dependent ADH (AdhE) was routinely followed as NADH (0.5 mM)-dependent reduction of acetaldehyde (10 mM). The standard assay for purification of the dominant ALDH (also AdhE, bifunctional ALDH/ADH) was defined as coenzyme A (CoA)-dependent (0.2 mM) reduction of NAD^+^ (2 mM) with 2 mM acetaldehyde as substrate followed at 340 nm (6.22 mM^−1^ cm^−1^). Purification of the NADPH-dependent ADH (AdhB) as well as purification of the recombinant Adh0564-His and His-AdhA was routinely followed as NADPH (0.5 mM)-dependent reduction of acetaldehyde (10 mM).

For the further characterization of the enzymes, NAD(P)^+^ (2 mM)-dependent oxidation of different alcohols (ethanol, 1-propanol, 1-butanol, 2-propanol, 2-butanol, 2, 3-butanediol) at a concentration of 200 mM (AdhE) or 500 mM (AdhB, Adh0564-His, His-AdhA) in the presence or absence of CoA (0.2 mM) was measured. For the substrate specificity of the ADH domain, the oxidation of NAD(P)H (0.5 mM) in the presence of different aldehydes and ketones (acetaldehyde, propionaldehyde, isobutyraldehyde, butyraldehyde, phenylacetaldehyde, acetone, 2, 3-butanedione) at a concentration of 10 mM (AdhE, AdhB) or 50 mM (Adh0564-His, His-AdhA) was determined. NAD(P)^+^ (2 mM)-dependent oxidation of different aldehydes (acetaldehyde, propionaldehyde, isobutyraldehyde, butyraldehyde) at a concentration of 10 mM was measured in the presence of 0.2 mM CoA.

Measurements for the determination of the pH optimum were performed in buffer containing 25 mM of each morpholinoethanesulfonic acid (MES), morpholinopropanesulfonic acid (MOPS), Tris, 2-(cyclohexylamino)ethanesulfonic acid (CHES) (pH as indicated) using the standard assay for each enzyme. For determination of the temperature optimum, enzymes were preincubated at the given temperature for 5 min before starting the measurement of the enzyme activity at the given temperature with the standard assays for each enzyme in 50 mM Tris pH 7.5. The temperature optimum of His-AdhA was measured in buffer containing 25 mM of each MES, MOPS, Tris and CHES (pH 9.0). To determine enzyme thermostability, enzymes were incubated at the given temperature for times ranging from 8 to 180 min. Following the incubation, residual enzyme activity was determined with the standard assays at certain time points at 65 °C. The half-life of the enzyme was determined from plotting the enzyme activity over time.

### Cloning of *adh* genes

The adhE (Teth514_0627), adhB (Teth514_0653), adhA (Teth514_0654) and adh0564 (Teth514_0564) genes were amplified from chromosomal DNA of *Thermoanaerobacter* sp. strain X514 by PCR with the primers listed in Table 4. PCR products were cleaved with suitable restriction enzymes, ligated into the overexpression vector pET21a (Novagen), followed by transformation into chemically competent *E. coli* cells. After screening of resulting transformants for plasmids of appropriate size, inserts were sequenced to ensure gene integrity. The final plasmids used in this study contained a sequence encoding a hexahistidine (His)-tag at the C terminus for the plasmids pET21a_AdhB-His, pET21a_AdhE-His and pET21a_Adh0564-His and a His-tag at the N terminus for the plasmid pET21a_His-AdhA for purification of the recombinant proteins.

### Production and purification of recombinant proteins

The production and purification of recombinant proteins was performed under aerobic conditions. The plasmids pET21a_AdhB-His, pET21a_AdhE-His, pET21a_Adh0564-His and pET21a_His-AdhA were transformed into chemically competent *E. coli* BL21 (DE3) grown on Luria–Bertani (LB) plates supplemented with 100 µg mL^−1^ ampicillin (Amp) at 37 °C. 5 mL precultures of the transformants were used to inoculate 500 mL LB medium containing 100 µg mL^−1^ Amp to an OD_600_ of 0.05, followed by incubation at 37 °C. Gene expression was induced at an OD_600_ of 0.6–0.8 by the addition of isopropyl-β-d-thiogalactopyranoside (IPTG) to a final concentration of 0.5 mM. After 3 h at 37 °C, the cultures were harvested, and cells were washed in buffer A (pH 7.5). To purify the recombinant ADHs, cells were resuspended in 2.5 mL buffer A (pH 7.5) including 0.5 mM phenylmethylsulfonyl fluoride (PMSF) and 0.1 mg mL^−1^ DnaseI and passed through a French Press at 110 MPa. Intact cells and cell debris were removed by centrifugation at 15,000×*g* for 10 min at 4 °C. The cell-free extract was incubated for 30 min with Ni^2+^–nitrilotriacetic acid (Ni^2+^–NTA) resin (column volume 2 mL; Quiagen, Hilden, Germany) equilibrated with 10 column volumes buffer D (50 mM NaH_2_PO_4_, 300 mM NaCl, 10 mM imidazole, pH 8.0). Nonspecifically bound protein was removed by washing with 10 column volumes buffer D containing 20 mM imidazole. Protein was eluted from the resin in six steps each with 2 mL buffer D containing 150 mM imidazole.

### Analytical methods

Protein concentrations were measured according to the method of Bradford [44]. Proteins were separated in 12% polyacrylamide gels after Laemmli [45] and stained with Coomassie brilliant blue G250. The molecular mass of the purified AdhE was determined by native gel electrophoresis under anaerobic conditions in the anaerobic chamber.

Protein subunits were cut from PAGE gels and sent for identification by liquid chromatography–mass spectrometry to Protein Analysis Group (Functional Genomics Center Zurich; Zurich).

The purified AdhE_His, AdhB-His, Adh0564-His and His-AdhA were used for immunization of a rabbit (Davids Biotechnologie, Regensburg, Germany) to generate antibodies for the immunological detection of the native proteins in *Thermoanaerobacter* sp. strain X514. For Western blot analysis, cells of *Thermoanaerobacter* sp. strain X514 were harvested in the late-exponential growth phase, washed and resuspended in buffer A (pH 7.5) including 0.5 mM phenylmethylsulfonyl fluoride (PMSF) and 0.1 mg mL^−1^ DnaseI and passed through a French Press at 110 MPa. Intact cells and cell debris were removed by centrifugation at 17,000×*g* for 30 min at 4 °C. 1 µg of purified proteins or cell-free extracts were separated by SDS-PAGE and proteins were transferred to a nitrocellulose membrane (Protran BA 83; GE Healthcare, Amersham, UK) according to the method of Towbin et al*.* [46]. Immunoblotting was performed with a 1:150,000 (anti-AdhB-His, anti-AdhE-His) and 1:90,000 (anti-Adh0564-His, anti-His-AdhA) dilution of the rabbit antiserum. Detection was carried out with a second alkaline phosphatase antibody and NBT/BCIP stock solution (Roche AG, Basel, Switzerland).

## Supplementary Information

**Additional file 1: Fig. S1.** Time dependence of natural DNA uptake by *Thermoanaerobacter* sp. strain X514. Subsamples were incubated for one hour at 65 °C with 1 µg ml^–1^ plasmid pIKM1, carrying a thermostable kanamycin resistance cassette, and then embedded in complex agar medium, and incubated in a N_2_/CO_2_ (80/20 *v/v*) -atmosphere for 5 days. Filled circles, OD_600;_ filled or open triangles, biological replicates of transformants per colony forming unit (CFU). **Fig. S2.** Affinity of the native AdhE (top chart) and the recombinant AdhE-His (bottom chart) for acetaldehyde, in the direction of ethanol formation. The assays were performed at 65 °C in cuvettes (Glasgerätebau Ochs, Bovenden-Lenglern, Germany) sealed by rubber stoppers. To the cuvettes, 1 mL 50 mM Tris buffer (pH 7.5) supplemented with 2 mM DTE and 4 µM resazurin, 0.5 mM NADH and 5.8 µg AdhE µg or 17.3 AdhE-His was added, and assays were started by the addition of acetaldehyde. **Fig. S3.** Affinity of the native AdhE for NADH, in the direction of ethanol formation. The assays were performed at 65 °C in cuvettes (Glasgerätebau Ochs, Bovenden-Lenglern, Germany) sealed by rubber stoppers. To the cuvettes, 1 mL 50 mM Tris buffer (pH 7.5) supplemented with 2 mM DTE and 4 µM resazurin, varying amounts of NADH, 5.8 µg AdhE and 10 mM acetaldehyde was added. **Fig. S4.** Affinity of the native AdhE for isobutyraldehyde, in the direction of isobutanol formation. The assays were performed at 65 °C in cuvettes (Glasgerätebau Ochs, Bovenden-Lenglern, Germany) sealed by rubber stoppers under a N_2_ atmosphere. The assay mixture contained 1 mL 50 mM Tris buffer (pH 7.5) supplemented with 2 mM DTE, 4 µM resazurin, 5.8 µg AdhE and 0.5 mM NADH. **Fig. S5.** Thermal stability of AdhE. The recombinant enzyme was incubated for up to 120 min at 65 °C (squares), 70 °C (triangles), 75 °C (diamonds) or 80 °C (crosses) before NADH-dependent acetaldehyde reduction was recorded at 65 °C. **Fig. S6.** Affinity of the native AdhB for NADPH. The assays were performed at 65 °C in cuvettes (Glasgerätebau Ochs, Bovenden-Lenglern, Germany) sealed with rubber stoppers, under a N_2_ atmosphere. The assay mixture contained 1 mL 50 mM Tris buffer (pH 7.5) supplemented with 2 mM DTE, 4 µM resazurin, 3.2 µg AdhB, 10 mM acetaldehyde and varying concentrations of NADPH. **Fig. S7.** Affinity of the native AdhB AdhB (top chart) and the recombinant AdhB-His (bottom chart) for acetaldehyde. The assays were performed at 65 °C in cuvettes (Glasgerätebau Ochs, Bovenden-Lenglern, Germany) sealed with rubber stoppers, under a N_2_ atmosphere. The assay mixture contained 1 mL 50 mM Tris buffer (pH 7.5) supplemented with 2 mM DTE, 4 µM resazurin, 3.2 µg AdhB or 15 µg AdhB-His, 0.5 mM NADPH and varying concentrations of acetaldehyde. **Fig. S8.** Thermal stability of AdhB-His. The recombinant enzyme was incubated for up to 180 min at 65 °C (squares), 80 °C (triangles), 85 °C (inverted triangles) or 90 °C (diamonds) before NADPH-dependent acetaldehyde reduction was recorded at 65 °C. **Fig. S9.** Affinity of the recombinant Adh0564 for NADPH, in the direction of ethanol formation. The assays were performed at 65 °C in cuvettes (Glasgerätebau Ochs, Bovenden-Lenglern, Germany) sealed by rubber stoppers. To the cuvettes, 1 mL 50 mM Tris buffer (pH 7.5) supplemented with 2 mM DTE and 4 µM resazurin, varying concentrations of NADPH and 9 µg Adh0564 was added, and assays were started by the addition of acetaldehyde. **Fig. S10.** Affinity of the recombinant Adh0564 for acetaldehyde, in the direction of ethanol formation. The assays were performed at 65 °C in cuvettes (Glasgerätebau Ochs, Bovenden-Lenglern, Germany) sealed by rubber stoppers. To the cuvettes, 1 mL 50 mM Tris buffer (pH 7.5) supplemented with 2 mM DTE and 4 µM resazurin, 0.5 mM NADH and 9 µg Adh0564 was added, and assays were started by the addition of acetaldehyde. **Fig. S11**. Affinity of the recombinant Adh0564 for isobutyraldehyde, in the direction of isobutanol formation. The assays were performed at 65 °C in cuvettes (Glasgerätebau Ochs, Bovenden-Lenglern, Germany) sealed by rubber stoppers under a N_2_ atmosphere. The assay mixture contained 1 mL 50 mM Tris buffer (pH 7.5) supplemented with 2 mM DTE, 4 µM resazurin, 16.4 µg Adh0564 and 0.5 mM NADH. **Fig. S12.** Affinity of the recombinant AdhA for NADPH, in the direction of ethanol formation. The assays were performed at 65 °C in cuvettes (Glasgerätebau Ochs, Bovenden-Lenglern, Germany) sealed by rubber stoppers. To the cuvettes, 1 mL 50 mM Tris buffer (pH 7.5) supplemented with 2 mM DTE and 4 µM resazurin, varying amounts of NADH, 19.8 µg AdhA and 10 mM acetaldehyde was added. **Fig. S13.** Affinity of the recombinant AdhA for acetaldehyde, in the direction of ethanol formation. The assays were performed at 65 °C in cuvettes (Glasgerätebau Ochs, Bovenden-Lenglern, Germany) sealed by rubber stoppers. To the cuvettes, 1 mL 50 mM Tris buffer (pH 7.5) supplemented with 2 mM DTE and 4 µM resazurin, 0.5 mM NADH and 18.5 µg AdhA was added, and assays were started by the addition of acetaldehyde. **Fig. S14.** Affinity of the recombinant AdhA for isobutyraldehyde, in the direction of isobutanol formation. The assays were performed at 65 °C in cuvettes (Glasgerätebau Ochs, Bovenden-Lenglern, Germany) sealed by rubber stoppers under a N_2_ atmosphere. The assay mixture contained 1 mL 50 mM Tris buffer (pH 7.5) supplemented with 2 mM DTE, 4 µM resazurin, 50 µg AdhA and 0.5 mM NADH. **Fig. S15.** Thermal stability of Adh0564-His. The recombinant enzyme was incubated for up to 120 min at 65 °C (squares), 70 °C (triangles), 80 °C (inverted triangles), 85 °C (diamonds) or 90 °C (filled circles) before NADPH-dependent acetaldehyde reduction was recorded at 65 °C. **Fig. S16.** Thermal stability of His-AdhA. The recombinant enzyme was incubated for up to 20 min at 65 °C (squares) or 65 °C (triangles) before NADPH-dependent acetaldehyde reduction was recorded at 65 °C. **Table S1.** Co-purification of the major NADH-dependent aldehyde dehydrogenase AdhE of *Thermoanaerobacter* sp. Strain X514 with the major NADH-dependent ADH (Table 1). NADH-dependent ALDH activity was measured as in 50 mM Tris buffer (pH 7.5) supplemented with 2 mM DTE and 4 µM resazurin as coenzyme A—(0.2 mM) and NAD^+^ (2 mM)—dependent oxidation of acetaldehyde (2 mM) at 340 nm 65 °C. One unit represents one µmol of acetaldehyde oxidized per minute. **Table S2.** Oligonucleotides developed and used in this study.

## Data Availability

Not applicable.

## Abbreviations

ADH: Alcohol dehydrogenase
ALDH: Aldehyde dehydrogenase
Amp: Ampicillin
AOR: Aldehyde ferredoxin oxidoreductase
CHES: 2-(Cyclohexylamino)ethanesulfonic acid
CoA: Coenzyme A
DTE: Dithioerythritol
IPTG: Isopropyl-β-d-thiogalactopyranoside
LC–MS: Liquid chromatography–mass spectrometry
MES: Morpholineethanesulfonic acid
MOPS: Morpholinepropanesulfonic acid
Ni^2^^+^–NTA: Ni^2^^+^–nitrilotriacetic acid
OD_600_: Optical density at 600 nm
PMSF: Phenylmethylsulfonyl fluoride
qRT-PCR: Quantitative real-time polymerase chain reaction
SDS-PAGE: Sodium dodecyl sulfate polyacrylamide gel electrophoresis
