# Supplementary material for: Alcohol dehydrogenases AdhE and AdhB with broad substrate ranges are important enzymes for organic acid reduction in Thermoanaerobacter sp. strain X514
Source: Biotechnol Biofuels. 2021 Sep 25;14:187. doi: 10.1186/s13068-021-02038-1 (PMC8466923; doi:10.1186/s13068-021-02038-1)
Supplement: Supplementary file 1 — Additional file 1: Fig. S1. Time dependence of natural DNA uptake by Thermoanaerobacter sp. strain X514. Subsamples were incubated for one hour at 65 °C with 1 µg ml–1 plasmid pIKM1, carrying a thermostable kanamycin resistance cassette, and then embedded in complex agar medium, and incubated in a N2/CO2 (80/20 v/v) -atmosphere for 5 days. Filled circles, OD600; filled or open triangles, biological replicates of transformants per colony forming unit (CFU). Fig. S2. Affinity of the native AdhE (top chart) and the recombinant AdhE-His (bottom chart) for acetaldehyde, in the direction of ethanol formation. The assays were performed at 65 °C in cuvettes (Glasgerätebau Ochs, Bovenden-Lenglern, Germany) sealed by rubber stoppers. To the cuvettes, 1 mL 50 mM Tris buffer (pH 7.5) supplemented with 2 mM DTE and 4 µM resazurin, 0.5 mM NADH and 5.8 µg AdhE µg or 17.3 AdhE-His was added, and assays were started by the addition of acetaldehyde. Fig. S3. Affinity of the native AdhE for NADH, in the direction of ethanol formation. The assays were performed at 65 °C in cuvettes (Glasgerätebau Ochs, Bovenden-Lenglern, Germany) sealed by rubber stoppers. To the cuvettes, 1 mL 50 mM Tris buffer (pH 7.5) supplemented with 2 mM DTE and 4 µM resazurin, varying amounts of NADH, 5.8 µg AdhE and 10 mM acetaldehyde was added. Fig. S4. Affinity of the native AdhE for isobutyraldehyde, in the direction of isobutanol formation. The assays were performed at 65 °C in cuvettes (Glasgerätebau Ochs, Bovenden-Lenglern, Germany) sealed by rubber stoppers under a N2 atmosphere. The assay mixture contained 1 mL 50 mM Tris buffer (pH 7.5) supplemented with 2 mM DTE, 4 µM resazurin, 5.8 µg AdhE and 0.5 mM NADH. Fig. S5. Thermal stability of AdhE. The recombinant enzyme was incubated for up to 120 min at 65 °C (squares), 70 °C (triangles), 75 °C (diamonds) or 80 °C (crosses) before NADH-dependent acetaldehyde reduction was recorded at 65 °C. Fig. S6. Affinity of the native AdhB for NADPH. The assays were p [file 13068_2021_2038_MOESM1_ESM.docx]

**Supplementary information for**

Alcohol dehydrogenases AdhE and AdhB with broad substrate ranges are important enzymes for organic acid reduction in *Thermoanaerobacter* sp. strain X514

Lisa Hitschler^2^,^1^, Laura Sofie Nissen^3^, Michelle Kuntz^4,1^, Mirko Basen^3, 1*^

^1^ Molecular Microbiology & Bioenergetics, Institute of Molecular Biosciences, Johann Wolfgang Goethe University Frankfurt/Main, Max-von-Laue Str. 9, 60438 Frankfurt/Main, Germany

^2^ Current address: Department of Membrane Biochemistry, Life and Medical Sciences (LIMES) Institute, University of Bonn, Carl-Troll-Straße 31, 53115 Bonn, Germany

^3^ Current address: Microbiology, Institute of Biological Sciences, University of Rostock, Albert-Einstein Str. 3, D-18059, Rostock, Germany

^4^ Current address: Interfaculty Institute for Microbiology and Infection Medicine Tübingen, University of Tübingen, Auf der Morgenstelle 24,72076 Tübingen, Germany

*Address correspondence to Mirko Basen ([mirko.basen@uni-rostock.de](mailto:mirko.basen@uni-rostock.de))

Table S1 Co-purification of the major NADH-dependent aldehyde dehydrogenase AdhE of *Thermoanaerobacter* sp. Strain X514 with the major NADH-dependent ADH (Table 1). NADH-dependent ALDH activity was measured as in 50 mM Tris buffer (pH 7.5) supplemented with 2 mM DTE and 4 µM resazurin as coenzyme A - (0.2 mM) and NAD^+^ (2 mM) - dependent oxidation of acetaldehyde (2 mM) at 340 nm 65°C. One unit represents one µmol of acetaldehyde oxidized per minute.

| **Purification**  **Step** | **Total protein**  **[mg]** | **Specific activity**  **[U mg^-1^]** | **Total activity**  **[U]** | **Yield**  **[%]** | **Purification**  **[fold]** |
| --- | --- | --- | --- | --- | --- |
| Crude extract | 1423 | 0.1 | 167 | 100.0 | 1.0 |
| Cytoplasm | 1196 | 0.1 | 165 | 98 | 1.2 |
| Q-Sepharose | 877 | 0.3 | 297 | 177 | 2.9 |
| (NH_4_)_2_SO_4_ precipitation | 511 | 0.7 | 334 | 200 | 5.6 |
| Phenyl-Sepharose | 220 | 1.6 | 352 | 211 | 13.6 |
| Superose6 | 2.8 | 9.7 | 27.3 | 16.3 | 82.4 |

Table S2 Oligonucleotides developed and used in this study.

| **Oligonucleotide** | **Sequence (5'-3')** | **Use** |
| --- | --- | --- |
| MK_058 | GAACGTGAAGGCGATAGTTG | qRT-PCR *Teth514_1380* |
| MK_059 | GCCGTTCCATAAGTTGGTAG | qRT-PCR *Teth514_1380* |
| MK_60 | AGGGCCTGACTTTCCTAC | qRT-PCR *Teth514_0010* |
| MK_061 | CGTCTTACCGTGGTGTTC | qRT-PCR *Teth514_0010* |
| MK_062 | ATTTGAGAAGGGCGAGGTC | qRT-PCR Teth514_1306 |
| MK_063 | TGGAGTTGGAACCCTCATC | qRT-PCR Teth514_1306 |
| LiH001 | ACGCCGGCTATAACTCCTAC | qRT-PCR Teth514_0627 |
| LiH002 | AAAACTGTCGGCATCCTCAA | qRT-PCR Teth514_0627 |
| LiH003 | TCGAAAATTTCCAGCCTGCC | qRT-PCR Teth514_0653 |
| LiH004 | CGCGTTGTTGTACCAGCTAT | qRT-PCR Teth514_0653 |
| LiH005 | TACCGAAGCTGCCACTACTC | qRT-PCR Teth514_0654 |
| LiH006 | CAACCTCACTGCCTCTTTCG | qRT-PCR Teth514_0654 |
| LiH027 | CTCATGACTGCGCAAAAGGG | qRT-PCR Teth514_1935 |
| LiH028 | TGCACTATTCCAACCACCACA | qRT-PCR Teth514_1935 |
| LiH029 | GGACTTTACCACCATTGCCG | qRT-PCR Teth514_1882 |
| LiH030 | ATTCAGCAAAAGCGCCATCC | qRT-PCR Teth514_1882 |
| LiH031 | TAGCAATTGTAACAGGCGGC | qRT-PCR Teth514_1808 |
| LiH032 | GCTCCTTCTTTTGCAAGCTCT | qRT-PCR Teth514_1808 |
| LiH033 | GTAGCGTCACCTTACAGCGT | qRT-PCR Teth514_0564 |
| LiH034 | AGGGTCATTTACAGCAGCGG | qRT-PCR Teth514_0564 |
| LiH035 | GGAAGTGAAGCGAGTGATGC | qRT-PCR Teth514_0241 |
| LiH036 | ACACCACAGGCCGTTTGATA | qRT-PCR Teth514_0241 |
| LiH037 | AGTGGCTGTAGGTGGAGGAA | qRT-PCR Teth514_0145 |
| LiH038 | GGTTTATGGGCGGCGGATTA | qRT-PCR Teth514_0145 |
| LiH009a | AATCATATGCACCATCACCATCACCATTGGGAAACTAAAATAAATCCCACCAAG | Cloning of pET21a_His_AdhA |
| LiH010a | AATCTCGAGTTAAGAGATTGGGTTAAGAGATTCTTCATAAATC | Cloning of pET21a_His_AdhA |
| LiH013 | AATCATATGAAAGGTTTTGCAATGCTCAGTA | Cloning of  pET21a_AdhB-His |
| LiH014 | AATGCGGCCGCTTAATGGTGATGGTGATGGTGTGCTAATATTACAACAGGTTTGATT | Cloning of  pET21a_AdhB-His |
| LiH017 | AATCATATGCCTACCTTATTACAAGAAAAA | Cloning of  pET21a_AdhE-His |
| LiH018 | AATGCGGCCGCTTAATGGTGATGGTGATGGTGTTCTCCATAGGCTTTTCTATATATTTC | Cloning of  pET21a_AdhE-His |
| LiH023a | AATCATATGTGGGAAACAAAAATAAATCCAAATAAGG | Cloning of pET21a_Adh0564-His |
| LiH024a | AATCTCGAGTTAATGGTGATGGTGATGGTGGAAAGATTCTTCATAAATCTTGGCAATAACAGC | Cloning of pET21a_Adh0564-His |

###

### Fig. S1 Time dependence of natural DNA uptake by *Thermoanaerobacter* sp. strain X514. Subsamples were incubated for one hour at 65°C with 1 µg ml^–1^ plasmid pIKM1, carrying a thermostable kanamycin resistance cassette, and then embedded in complex agar medium, and incubated in a N_2_/CO_2_ (80/20 *v/v*) -atmosphere for 5 days. Filled circles, OD_600;_ filled or open triangles, biological replicates of transformants per colony forming unit (CFU).

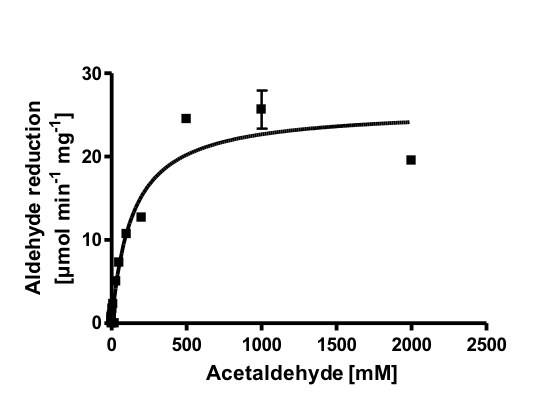


### Fig. S2 Affinity of the native AdhE (top chart) and the recombinant AdhE-His (bottom chart) for acetaldehyde, in the direction of ethanol formation. The assays were performed at 65°C in cuvettes (Glasgerätebau Ochs, Bovenden-Lenglern, Germany) sealed by rubber stoppers. To the cuvettes, 1 mL 50 mM Tris buffer (pH 7.5) supplemented with 2 mM DTE and 4 µM resazurin, 0.5 mM NADH and 5.8 µg AdhE µg or 17.3 AdhE-His was added, and assays were started by the addition of acetaldehyde.

###

**Fig. S3** Affinity of the native AdhE for NADH, in the direction of ethanol formation. The assays were performed at 65°C in cuvettes (Glasgerätebau Ochs, Bovenden-Lenglern, Germany) sealed by rubber stoppers. To the cuvettes, 1 mL 50 mM Tris buffer (pH 7.5) supplemented with 2 mM DTE and 4 µM resazurin, varying amounts of NADH, 5.8 µg AdhE and 10 mM acetaldehyde was added.

**Fig. S4** Affinity of the native AdhE for isobutyraldehyde, in the direction of isobutanol formation. The assays were performed at 65 °C in cuvettes (Glasgerätebau Ochs, Bovenden-Lenglern, Germany) sealed by rubber stoppers under a N_2_ atmosphere. The assay mixture contained 1 mL 50 mM Tris buffer (pH 7.5) supplemented with 2 mM DTE, 4 µM resazurin, 5.8 µg AdhE and 0.5 mM NADH.

**Fig. S5** Thermal stability of AdhE. The recombinant enzyme was incubated for up to 120 min at 65°C (squares), 70°C (triangles), 75°C (diamonds) or 80°C (crosses) before NADH-dependent acetaldehyde reduction was recorded at 65°C.

**Fig. S6** Affinity of the native AdhB for NADPH. The assays were performed at 65 °C in cuvettes (Glasgerätebau Ochs, Bovenden-Lenglern, Germany) sealed with rubber stoppers, under a N_2_ atmosphere. The assay mixture contained 1 mL 50 mM Tris buffer (pH 7.5) supplemented with 2 mM DTE, 4 µM resazurin, 3.2 µg AdhB, 10 mM acetaldehyde and varying concentrations of NADPH.

**Fig. S7** Affinity of the native AdhB (top chart) and the recombinant AdhB-His (bottom chart) for acetaldehyde. The assays were performed at 65 °C in cuvettes (Glasgerätebau Ochs, Bovenden-Lenglern, Germany) sealed with rubber stoppers, under a N_2_ atmosphere. The assay mixture contained 1 mL 50 mM Tris buffer (pH 7.5) supplemented with 2 mM DTE, 4 µM resazurin, 3.2 µg AdhB or 15 µg AdhB-His, 0.5 mM NADPH and varying concentrations of acetaldehyde.

**Fig. S8** Thermal stability of AdhB-His. The recombinant enzyme was incubated for up to 180 min at 65°C (squares), 80°C (triangles), 85°C (inverted triangles) or 90°C (diamonds) before NADPH-dependent acetaldehyde reduction was recorded at 65°C.

**Fig. S9** Affinity of the recombinant Adh0564 for NADPH, in the direction of ethanol formation. The assays were performed at 65°C in cuvettes (Glasgerätebau Ochs, Bovenden-Lenglern, Germany) sealed by rubber stoppers. To the cuvettes, 1 mL 50 mM Tris buffer (pH 7.5) supplemented with 2 mM DTE and 4 µM resazurin, varying concentrations of NADPH and 9 µg Adh0564 was added, and assays were started by the addition of acetaldehyde.

**Fig. S10** Affinity of the recombinant Adh0564 for acetaldehyde, in the direction of ethanol formation. The assays were performed at 65°C in cuvettes (Glasgerätebau Ochs, Bovenden-Lenglern, Germany) sealed by rubber stoppers. To the cuvettes, 1 mL 50 mM Tris buffer (pH 7.5) supplemented with 2 mM DTE and 4 µM resazurin, 0.5 mM NADH and 9 µg Adh0564 was added, and assays were started by the addition of acetaldehyde.

**Fig. S11** Affinity of the recombinant Adh0564 for isobutyraldehyde, in the direction of isobutanol formation. The assays were performed at 65 °C in cuvettes (Glasgerätebau Ochs, Bovenden-Lenglern, Germany) sealed by rubber stoppers under a N_2_ atmosphere. The assay mixture contained 1 mL 50 mM Tris buffer (pH 7.5) supplemented with 2 mM DTE, 4 µM resazurin, 16.4 µg Adh0564 and 0.5 mM NADH.

**Fig. S12** Affinity of the recombinant AdhA for NADPH, in the direction of ethanol formation. The assays were performed at 65°C in cuvettes (Glasgerätebau Ochs, Bovenden-Lenglern, Germany) sealed by rubber stoppers. To the cuvettes, 1 mL 50 mM Tris buffer (pH 7.5) supplemented with 2 mM DTE and 4 µM resazurin, varying amounts of NADH, 19.8 µg AdhA and 10 mM acetaldehyde was added.

**Fig. S13** Affinity of the recombinant AdhA for acetaldehyde, in the direction of ethanol formation. The assays were performed at 65°C in cuvettes (Glasgerätebau Ochs, Bovenden-Lenglern, Germany) sealed by rubber stoppers. To the cuvettes, 1 mL 50 mM Tris buffer (pH 7.5) supplemented with 2 mM DTE and 4 µM resazurin, 0.5 mM NADH and 18.5 µg AdhA was added, and assays were started by the addition of acetaldehyde.

**Fig. S14** Affinity of the recombinant AdhA for isobutyraldehyde, in the direction of isobutanol formation. The assays were performed at 65 °C in cuvettes (Glasgerätebau Ochs, Bovenden-Lenglern, Germany) sealed by rubber stoppers under a N_2_ atmosphere. The assay mixture contained 1 mL 50 mM Tris buffer (pH 7.5) supplemented with 2 mM DTE, 4 µM resazurin, 50 µg AdhA and 0.5 mM NADH.

**Fig. S15** Thermal stability of Adh0564-His. The recombinant enzyme was incubated for up to 120 min at 65°C (squares), 70°C (triangles), 80°C (inverted triangles), 85°C (diamonds) or 90°C (filled circles) before NADPH-dependent acetaldehyde reduction was recorded at 65°C.

**Fig. S16** Thermal stability of His-AdhA. The recombinant enzyme was incubated for up to 20 min at 65°C (squares) or 65°C (triangles) before NADPH-dependent acetaldehyde reduction was recorded at 65°C.
